# A brief history of how microscopic studies led to the elucidation of the 3D architecture and macromolecular organization of higher plant thylakoids

**DOI:** 10.1007/s11120-020-00782-3

**Published:** 2020-10-05

**Authors:** L. Andrew Staehelin, Dominick J. Paolillo

**Affiliations:** 1 Department of Molecular, Cellular and Developmental Biology, UCB 347, University of Colorado, Boulder, CO 80309-0347 USA; 2 Plant Biology Section, School of Integrative Plant Science, Cornell University, Ithaca, NY 14853 USA

**Keywords:** Chloroplasts, Electron microscopy, Electron tomography, Thylakoid model, Freeze-fracture electron microscopy, Atomic force microscopy

## Abstract

Microscopic studies of chloroplasts can be traced back to the year 1678 when Antonie van Leeuwenhoek reported to the Royal Society in London that he saw green globules in grass leaf cells with his single-lens microscope. Since then, microscopic studies have continued to contribute critical insights into the complex architecture of chloroplast membranes and how their structure relates to function. This review is organized into three chronological sections: During the classic light microscope period (1678–1940), the development of improved microscopes led to the identification of green grana, a colorless stroma, and a membrane envelope. More recent (1990–2020) chloroplast dynamic studies have benefited from laser confocal and 3D-structured illumination microscopy. The development of the transmission electron microscope (1940–2000) and thin sectioning techniques demonstrated that grana consist of stacks of closely appressed grana thylakoids interconnected by non-appressed stroma thylakoids. When the stroma thylakoids were shown to spiral around the grana stacks as multiple right-handed helices, it was confirmed that the membranes of a chloroplast are all interconnected. Freeze-fracture and freeze-etch methods verified the helical nature of the stroma thylakoids, while also providing precise information on how the electron transport chain and ATP synthase complexes are non-randomly distributed between grana and stroma membrane regions. The last section (2000–2020) focuses on the most recent discoveries made possible by atomic force microscopy of hydrated membranes, and electron tomography and cryo-electron tomography of cryofixed thylakoids. These investigations have provided novel insights into thylakoid architecture and plastoglobules (summarized in a new thylakoid model), while also producing molecular-scale views of grana and stroma thylakoids in which individual functional complexes can be identified.

## Introduction

Throughout history the introduction of new research techniques has led to significant advances in scientific knowledge. This has also been the case for investigations of the architecture of higher plant chloroplast membranes, where the introduction of new and better microscopes and analytical methods has improved the spatial resolution by ×5000, from ~ 1.0 μm to ~ 0.2 nm.

The first phase of chloroplast structure research coincided with the development of new light microscopes with better lenses. The second with the introduction of the electron microscope and the parallel development of improved specimen preparation methods. The latest phase encompasses the use of atomic force microscopy and the exploitation of electron tomography to analyze the structure of cryofixed chloroplasts and to create both high-resolution 3D models of thylakoid architecture and information about the macromolecular organization of thylakoid membranes.

### The classical light microscope era of chloroplast research defined the basic elements of chloroplast architecture, while new microscopes provide insights into thylakoid dynamics

**Fig. 1:**
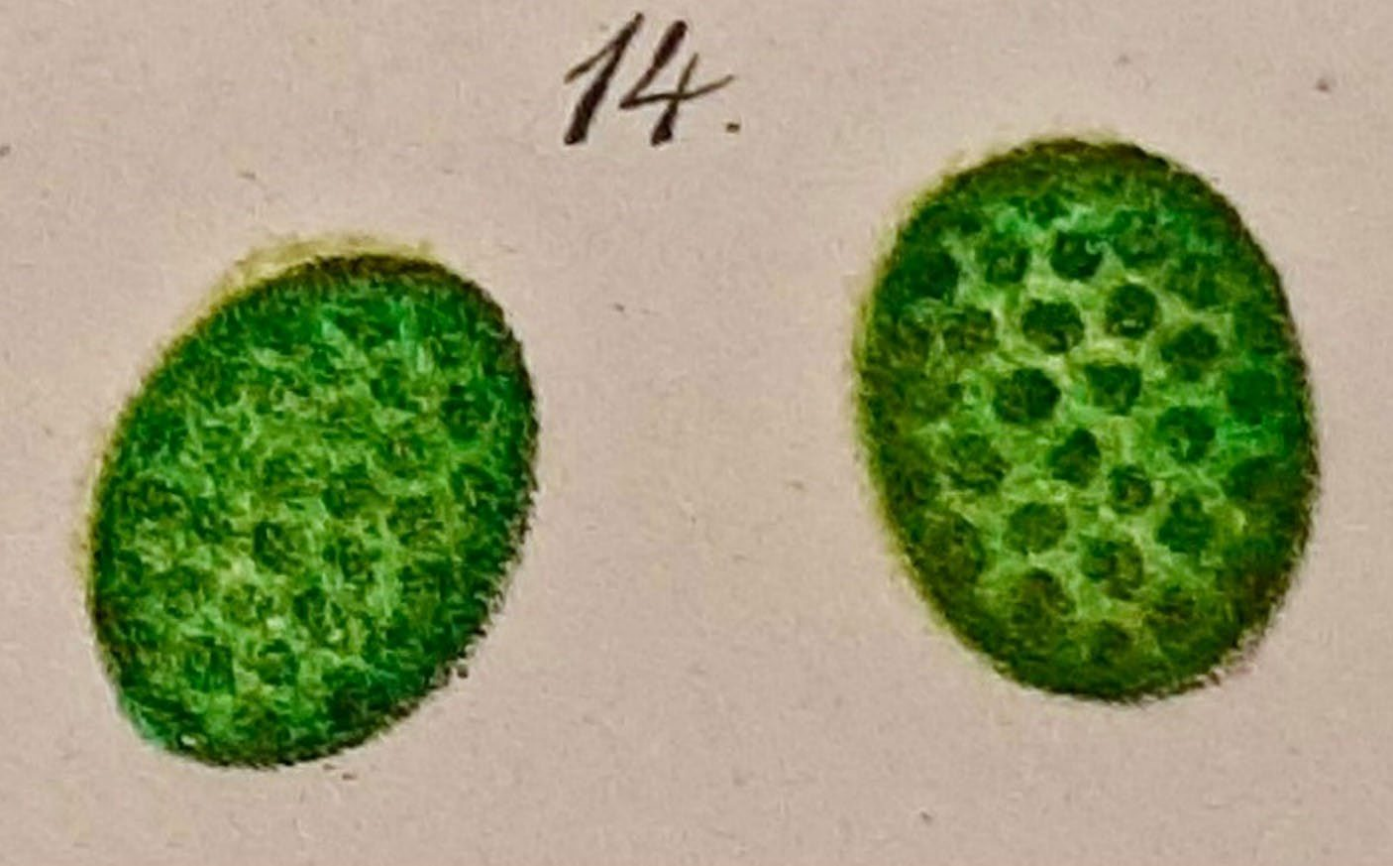
Chloroplast drawings of Arthur Meyer (1883) that were hand-colored by publisher. The images highlight the chloroplast grana he observed in leaf cells of *Acanthephippium*

Although the German botanist Hugo von Mohl (1837) is generally credited with the discovery and definitive description of the “Chlorophyllkörner”—chloroplast granules, the first reports of green granules were published much earlier. For example, in 1678 Antonie van Leeuwenhoek, a draper by trade and a microscope lens maker extraordinaire, wrote a letter to the Royal Society London (cited in Weier 1938) in which he described green globules in grass leaf cells that he saw with his single-lens microscope (the magnification of a van Leeuwenhoek microscope in the Utrecht Museum is ~ 275×). One hundred years later, similar findings were published by Comparetti in 1791 and Sprengel in 1802 (cited in Weier 1938). Both observed green grains that moved around in plant cells. In 1818, the green pigment of leaves was given the name “chlorophyll” by Pelletier and Caventou (cited in Loomis, 1960). The same year that von Mohl (1837) published his detailed description of chloroplasts, Meyen (1837) noted the presence of dark spots within the chlorophyll-containing bodies. A quarter century later, Sachs (1862) and other microscopists reported that the chlorophyll within the green bodies was present in the dark spots/granules. An accurate description of these green pigmented granules surrounded by colorless material, however, was only published another two decades later by Meyer (1883), who named them “grana” (Fig. 1). Pringsheim (1882) gave us the word”stroma” to describe the residual structures left after extraction of the chlorophyll. The name was subsequently transferred by Schimper (1885) to the colorless substance surrounding the grana, and this definition is still in use today.

Recognizing that the Chlorophyllkörner were part of a group of related organelles Schimper (1883) proposed the following nomenclature for the family of “Plastiden” (Greek word Plastikos, meaning formed or molded). The Chlorophyllkörner became “Chloroplastiden”, the colorless, starch-containing plastids “Leukoplastiden” and the red/orange-colored plastids “Chromoplastiden.” This list was expanded by Strugger (1950) to include the “proplastids” of dividing cells.

The development of chloroplasts from colorless granules during light-induced greening of immature leaf cells was first described by Sachs (1859). This was followed by multiple reports of chloroplast division by fission (e.g., Meyer 1883; Schimper 1885), which led to the theory of plastid continuity.

The presence of a chloroplast surrounding membrane was postulated by Nägeli (1948), who referred to chloroplasts as “Bläschen”—small bubbles. Tschirch (1884), using *Elodea* as an experimental system, came to the conclusion that chloroplasts were encompassed by a membrane that was like the one surrounding cells. His ideas, however, were forcefully attacked by Schmitz (1884), Meyer (1883) and Schimper (1885), which led to the chloroplast envelope membrane concept being largely forgotten until Wieler (1936) concluded from his own and other researcher’s data that chloroplasts were enclosed in a semipermeable membrane.

During the first decades of the twentieth century the introduction of the concept of colloid chemistry to biology led to the claim that the grana were fixation (coagulation) artifacts and that chloroplasts had a homogenous interior. This claim was supported by images of chloroplasts that were devoid of internal structures when viewed using dark field microscopy (Price 1914). The interior of chloroplasts was postulated to be either a colloid or a hydrogel within which the chlorophyll was suspended in a lipoidal phase (e.g., Schürhoff 1924). This idea lived on in textbooks until the mid 1930s (e.g., Küster 1935).

The era of hydrogel/colloid filled chloroplasts came to sudden end when in the same year Doutreligne (1935), using monochromatic red light photography, visualized grana in the chloroplasts of transparent leaf cells of water plants in their natural growth medium. In his classical study of the interior structures of chloroplasts, Heitz (1936) was able to resolve grana in living cells of all classes of higher plants (~ 180 different species) using a high numerical apochromatic objective and blue light illumination. Other observations reported in that paper were (1) that the size of grana was variable (~ 0.3 to ~ 1.0 μm) with shade plant chloroplasts displaying larger diameter grana than sun plants, (2) that the grana were not round granules but cylinders with a layered substructure as evidenced by their birefringence, and (3) that the grana cylinders were oriented vertically to the plane of the cell walls. In the same year, Weier (1936) described how the distribution of grana within chloroplasts could be manipulated experimentally.

By comparing face-on and side views of the plate-like chloroplasts of *Mugeotia* and other chloroplasts, Menke (1934) discovered that grana exhibited negative birefringence in side views and were isotropic in face-on views. Since this birefringence disappeared upon infusion of the cells with glycerol, he concluded that the grana exhibited form birefringence. His chloroplast model depicts grana as integral parts of longer lamina that spanned the length of the chloroplasts (Menke 1940). Based on all of the studies listed above, available chemical and physical properties of known chloroplast molecules, and his own quantitative birefringence studies, Frey-Wyssling (1938) calculated a vertical granum repeat distance of ~ 30 nm and proposed that this repeat was comprised of a lipidic bilayer and a protein layer. While the molecular details of this model are outdated, the ~ 30 nm repeat is remarkably close to the repeat of stacked grana membranes determined by electron microscopy (e.g., Kirchhoff et al. 2011).

### Laser confocal and 3D-structured illumination microscopy have provided detailed insights into chloroplast dynamics

Although, as discussed in the following sections, electron microscopic techniques have been the principal tool for advancing our understanding of thylakoid architecture and functional organization during the past 80 years, light microscopes have continued to be used to investigate dynamic changes of chloroplasts in living cells to address questions that cannot be answered by electron microscope analyses. For example, laser confocal microscopy can not only produce high-resolution fluorescence microscopy images of living chloroplasts but can also be used to perform fluorescence recovery after photobleaching experiments (Mullineaux 2004). With these techniques, Johnson et al. (2011) have investigated the changes in structural reorganization of photosynthetic membranes during the formation of the photoprotective state. Their results demonstrated that during this process the LHCII proteins dissociate from PSII and then form aggregates. These observations were confirmed by correlative analyses of freeze-fractured thylakoid membranes (see third section of this review for details on the freeze-fracture technique).

Most recently, three-dimensional structured illumination microscopy (3D-SIM) has been introduced to photosynthesis research by Iwai et al. (2018) to explore the optimal imaging conditions for investigating the dynamics of thylakoid membranes in living *Arabidopsis* cells with sub-diffraction resolution (100–300 nm in lateral dimension and 280–350 nm in vertical dimension). They tested whether 3D-SIM can be used to characterize the structural responses of thylakoids in intact leaves exposed to either far red (FR) or blue (BL) light for 2 h. They observed that these conditions induce the dephosphorylation/phosphorylation of LHCII, which cause changes in the diameter of the grana, from ~ 370 nm in FR versus ~ 304 nm in BL. Similar observations were made by Kyle et al. (1983) using the technique of freeze-fracture electron microscopy.

## Classical transmission electron microscopy (TEM) led to the formulation of the helical model of thylakoid architecture

In this section we review the progression of TEM observations that led to the formulation of the generally accepted helical model of granum structure.

**Fig. 2:**
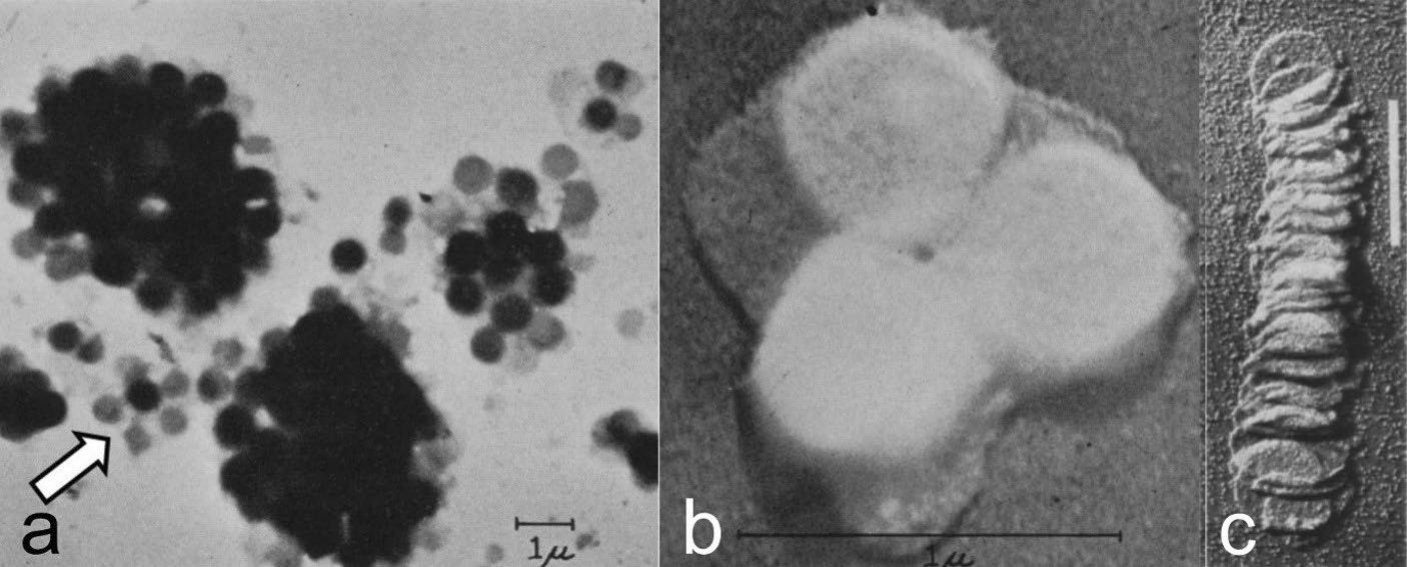
Early electron micrographs of isolated chloroplast membranes. **a** Clusters of grana from individual, disrupted spinach chloroplasts air-dried on grid. The arrow points to a small, separated cluster of grana that appear interconnected by membranes (from Granick and Porter 1947). **b** Cluster of three, round, gold-shadowed grana “stacks” at high magnification (from Granick and Porter 1947). **c** Disassembled and gold-shadowed, disrupted granum of the shade plant *Aspidistra* that resembles a toppled stack of coins. From Steinmann (1952). Bar 1.0 mm

When Kausche and Ruska (1940) were exploring the usefulness of the first electron microscope for biological studies, one of their test specimens were chloroplasts infected with the tobacco mosaic virus. While demonstrating the rod-like nature of the viruses, their images of disrupted chloroplasts also showed round, face-on views of grana. These grana were column like and, as shown subsequently by Granick and Porter (1947) and Steinman (1952), exhibited a layered substructure (Fig. 2a–c). Indeed, the micrograph depicted in Fig. 2c became a classic in the photosynthesis literature, because it illustrated unambiguously that the grana were composed of disks that could be separated like a stack of coins. Evidence for the interconnectedness of grana is highlighted by the arrow in Fig. 2a.

**Fig. 3:**
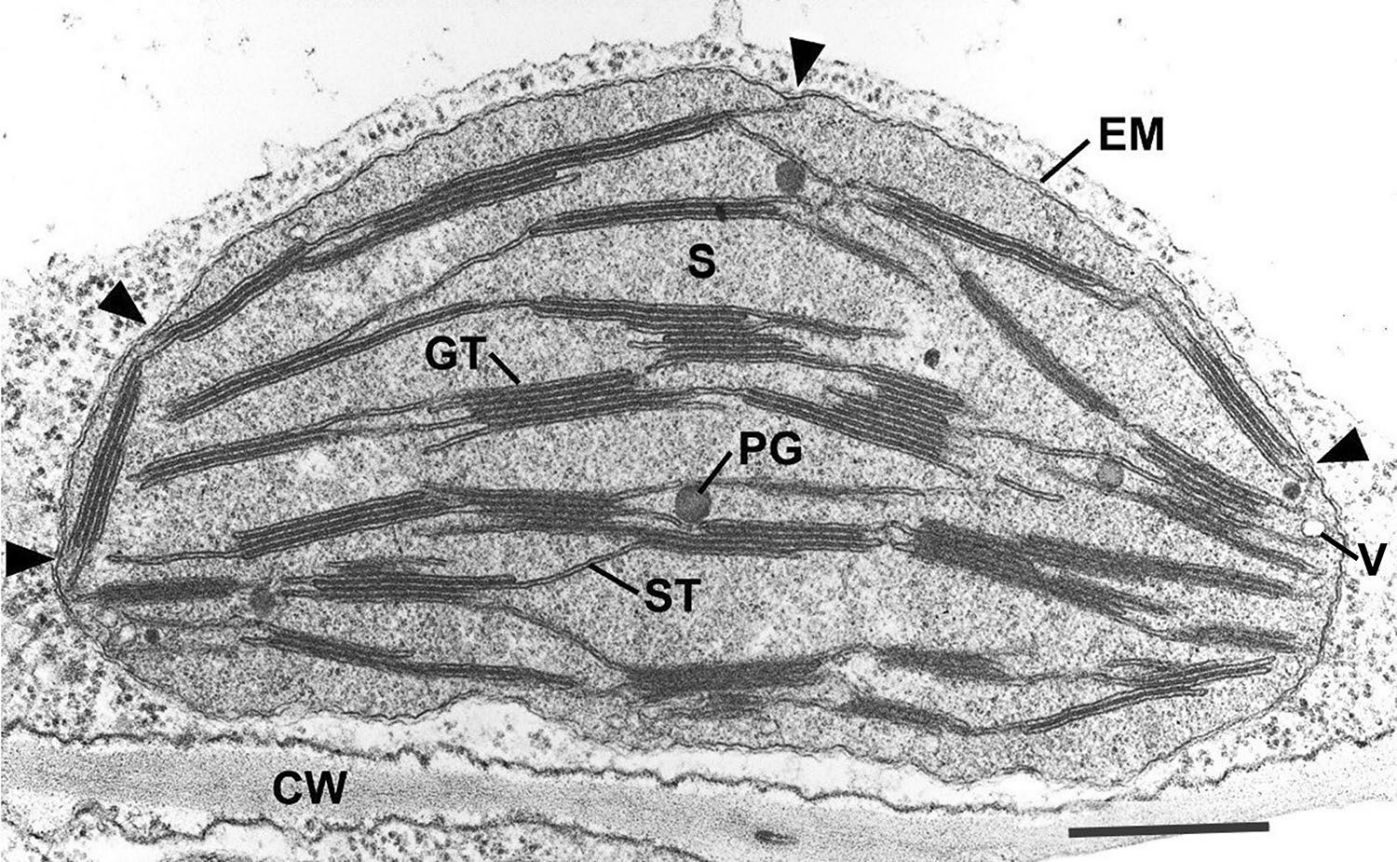
Thin section electron micrograph of a chemically fixed chloroplast in a young tobacco leaf. The chloroplast lies flat against the plasma membrane and the cell wall (CW) and presents a more or less elliptical outline. The stacked grana thylakoids (GT) are interconnected by non-stacked stroma thylakoids (ST). Stroma (S) surrounds the membranes, and the lightly stained regions of the stroma indicates the presence of DNA. Because this chloroplast was still growing, when it was fixed for TEM analysis, the grana stacks vary in height and have irregular margins. A few plastoglobules (PG) lie adjacent to stroma thylakoids. Two envelope membranes (EM) form the boundary layer of the chloroplast. The arrowheads point to contact sites between thylakoid membranes and the inner envelope membrane. Such sites are frequently seen in growing chloroplasts and most likely represent sites of galactolipid transfer from the lipid-synthesizing inner envelope membrane to the growing thylakoid membranes. A small vesicle (V) is seen close to the inner envelope membrane. They are also seen infrequently. From Staehelin (1986); Bar 0.5 mm

**Fig. 4:**
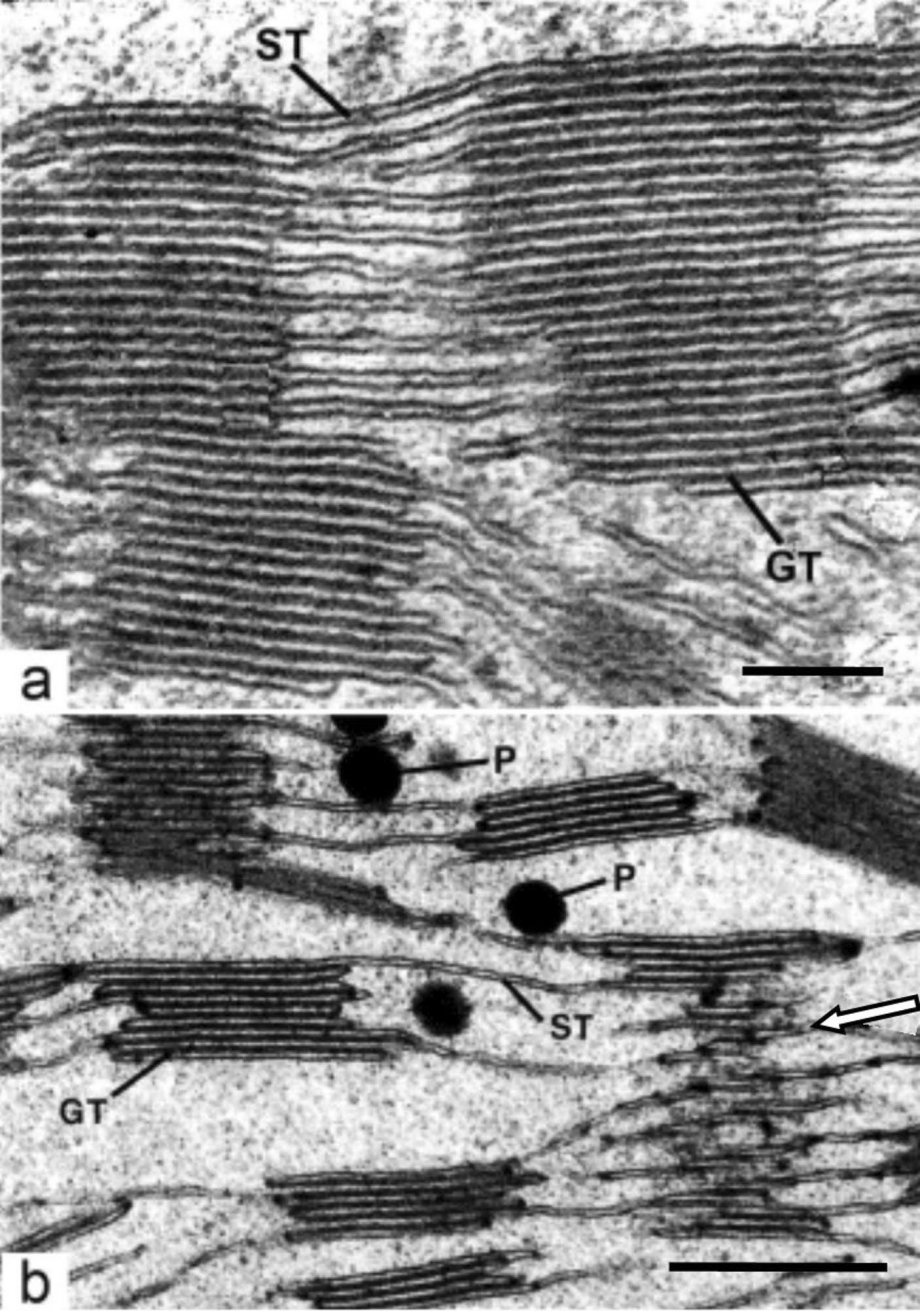
Higher magnification micrographs of grana (GT) and stroma thylakoids (ST) in corn and spinach chloroplasts. **a** Corn chloroplast. In this cross-sectional view, the continuity between stacked grana thylakoids and non-stacked stroma thylakoids is clearly seen, as is the partial overlap between two grana. In many instances, the grana and stroma thylakoids appear to be connected to each other either through bifurcated stroma thylakoids or through membranes that continue in a planar manner between the two types of thylakoid. Configurations of this kind dominated early models of membrane architecture. The ratio of grana thylakoids to stroma thylakoids approximates 2:1. **b** Grana thylakoids and stroma thylakoids of a spinach chloroplast. The tangentially sectioned granum seen on the lower right side illustrates the angle (arrow) between the different planes of the grana and stroma membranes. The latter are also evenly spaced (cf. Fig. 7). Plastoglobuli (P). **a** From Paolillo and Falk (1966); **b** From Staehelin (1986). **a**, **b** Bars 0.5 mm

The development of ultramicrotomes (reviewed in Porter and Blum 1953) constituted the next major technical advance in TEM. This advance required not only the construction of microtomes capable of producing sections thin enough (10–20 nm) to be viewed in a TEM, but simultaneously the development of improved chemical fixation and staining protocols and the discovery of embedding resins such as methacrylate to replace paraffin, and of glass knives to replace specially sharpened steel blades. As anticipated from images like that in Fig. 2c, each granum was visualized in cross-sections as a multilayered cylinder. In mesophyll cells the chloroplasts lie close to the cell membrane and present a more or less elliptical outline in a plane of sectioning at right angles to the cell wall (Fig. 3). In that plane the stacking of the grana membranes is clearly seen, whereas in sections parallel to the thylakoid membranes the grana appear mostly circular (not shown). Hence the idealization of a granum as a cylinder. As illustrated in Fig. 4a, b the number of membranes in grana stacks is highly variable, leading to authors often referring to the “height” of a granum as a measure of the number of stacked membranes. The highly curved, non‐appressed edges of the grana are called “margins”.

Less appreciated is the fact that stroma thylakoids form contact sites with the inner envelope membrane (IEM) (Fig. 3, arrowheads). At the same time (not shown), the outer envelope membrane forms contact sites with membranes of the endoplasmic reticulum (ER) (Wang and Benning 2012). Contact sites between the ER and other membrane systems are a common feature of plant cells (Staehelin 1997) and such sites have been shown to mediate the molecular transfer of lipids via a lipid-hopping mechanism (Lev 2010). In developing chloroplasts, the contact sites are formed mostly between the margins of the thylakoids and the IEM and are randomly distributed along the envelope membrane adjacent to the cytoplasm (Fig. 3; Charuvi et al. 2012; Liang et al. 2018; Hertle et al. 2020). As the thylakoids grow larger they orient parallel to the plasma membrane, while their marginal contact sites become concentrated in the chloroplast poles (Liang et al. 2018; Hertle et al. 2020). Functionally, the thylakoid-IEM contact sites appear to be responsible for transferring uncharged galactolipids (mono- and di-galactosyldiacylglycerols) from their site of synthesis, the chloroplast envelope membranes (Kobayashi et al. 2007), to the thylakoids.

Wild type chloroplasts also contain small (~ 70 nm) vesicles (Fig. 3; Wise and Hoober 2007; Liang et al. 2018; Hertle et al. 2020). These vesicles have garnered a lot of attention by molecular biologists interested in identifying chloroplast homologs of proteins known to mediate cytoplasmic vesicle formation and transport (Lindquist and Aronson 2018; Mechela et al. 2019). As reported by Hertle et al. 2020), the chloroplast Sec 14-like protein (CPSFL1) localizes to both chloroplast vesicles and to infoldings of the IEM. These vesicles are needed for photoautotrophic growth. CPSFL1 binds phosphatidylinositolphosphates (PIPs) and phosphatidic acid (PA), but not uncharged galactolipids, and is capable of transferring these lipids between membranes. The *cpsfl1* mutant does not produce vesicles and forms sheet-like thylakoids that lack grana stacks. These mutant thylakoids span the width of the chloroplasts and form very distinct contact sites at the chloroplast poles. Another chloroplast mutant, *vpp1* is also unable to produce IEM-derived vesicles and exhibits a phenotype similar to cpsfl1 (Zhang and Sakamoto 2015). A recent cyro-electron microscopy study has produced high-resolution structures of VPP1 rings produced in vitro, which illustrate how VPP1 monomers interact with each other and how they bind membrane lipid molecules and thereby induce membrane curvature (Gupta et al. 2020). Together, these results support the hypothesis that chloroplast vesicles are responsible for the transport of PIPs and PA from the IEM to the thylakoids, whereas the IEM thylakoid contact sites serve the transfer of galactolipids to the thylakoids.

Based on the appearance of grana membranes in early TEM micrographs they were interpreted to be composed of closely appressed membrane-bound saccules, or vesicles, that were interconnected by non-stacked stroma thylakoids (Fig. 3; Steinman and Sjöstrand 1955). These saccules were given the name “thylakoids” by Menke (1962). Weier and coworkers (Weier 1961; Weier and Thomson 1962) referred to the intergranal membrane networks as fretworks and called the individual compartments frets. However, their nomenclature was never widely adopted, and the term stroma thylakoids is now the only name used.

Precise modeling of how the grana are joined to the stroma thylakoids depends on reproducible and accurate microscopic data. In hindsight the limitations of the quality of the early images of thin sections is clearly evident, and grounds for questioning their use to construct models of chloroplast membrane architecture. Nevertheless, early researchers assumed that individual sections imparted sufficient information to suggest models of how chloroplast membranes were organized (e.g., Hodge et al. 1955; Steinman and Sjöstrand 1955; Menke 1960; von Wettstein 1961; Eriksson et al. 1961; Sitte 1962).

**Fig. 5:**
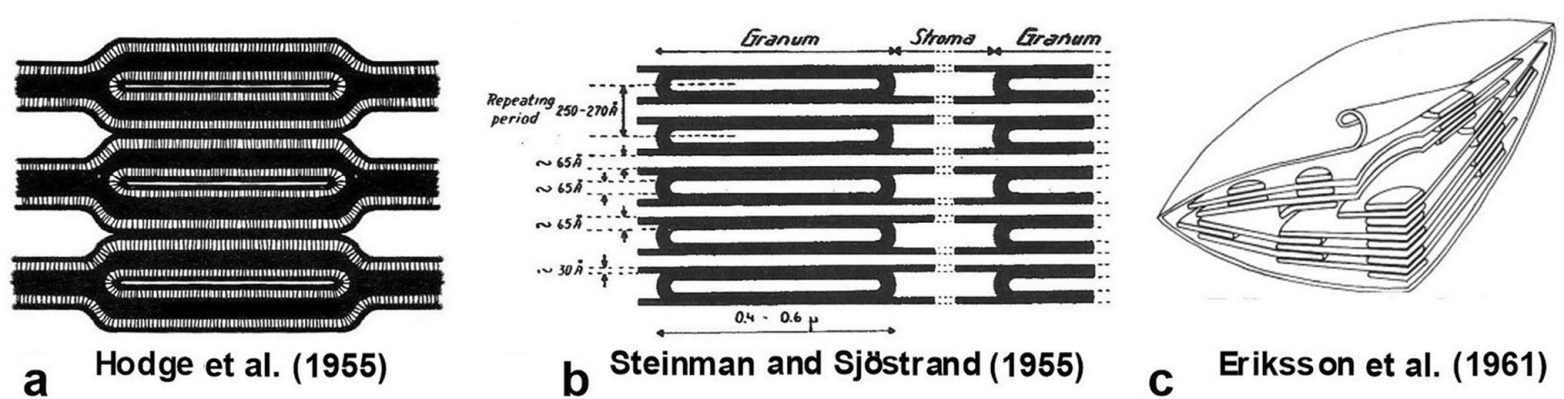
Early models of chloroplast membrane architecture. **a** The grana and stroma thylakoids of the Hodge et al. (1955) model are shown as connected through Y-shaped junctional regions. **b** The competing model of Steinman and Sjöstrand (1955) shows expansive stroma thylakoids and restricted grana thylakoids. Where the non-stacked stroma thylakoids enter the grana stack, they become grana thylakoids. **c** 3D chloroplast model of Eriksson et al. (1961) based on the premise that the thylakoids are arranged as in (**b**)

This led to the idea that grana thylakoids were uniformly connected to stroma thylakoids throughout the height of a granum stack (Fig. 5a, b), that the stroma thylakoids were broad sheets, and that they were continuous with, and orientated parallel to, the grana thylakoids. However, while the models of Hodge et al. (1955; Fig. 5a) and Steinman and Sjöstrand (1955; Fig. 5b) both showed sheet-like stroma thylakoids, with a 2:1 ratio of grana thylakoids to stroma thylakoids, they differed in how they depicted the grana-stroma membrane transition regions.

The two models can best be distinguished by considering two vertically successive thylakoids in a section of a granum stack. In the Hodge et al. (1955) model two successive grana thylakoids are shown as joined to each other and to a stroma thylakoid by what can be characterized as a horizontal Y-shaped, bifurcation configuration, with the stem of the Y continuous with the stroma thylakoid at both edges of the granum. The conjunction of adjacent grana thylakoids was given as uniform throughout the height of a stack. In the alternative model (Steinmann and Sjöstrand 1955; Mühlethaler and Frey-Wyssling 1959; Menke 1960; von Wettstein 1960; Wehrmeyer 1961), one of the two grana thylakoids is shown as being limited to the grana stack, while the other becomes a stroma thylakoid on both sides of the grana stack. Although both the Hodge et al. (1955) and the Steinman and Sjöstrand (1955)-type membrane configurations could be discerned in the same electron micrographs (e.g., Fig. 4a), most authors at that time conveniently overlooked the membrane configurations that did not conform to their model. As pointed out by Paolillo and Reighard (1967), what was missing from those earlier studies was a rigorous consideration of the third dimension.

**Fig. 6:**
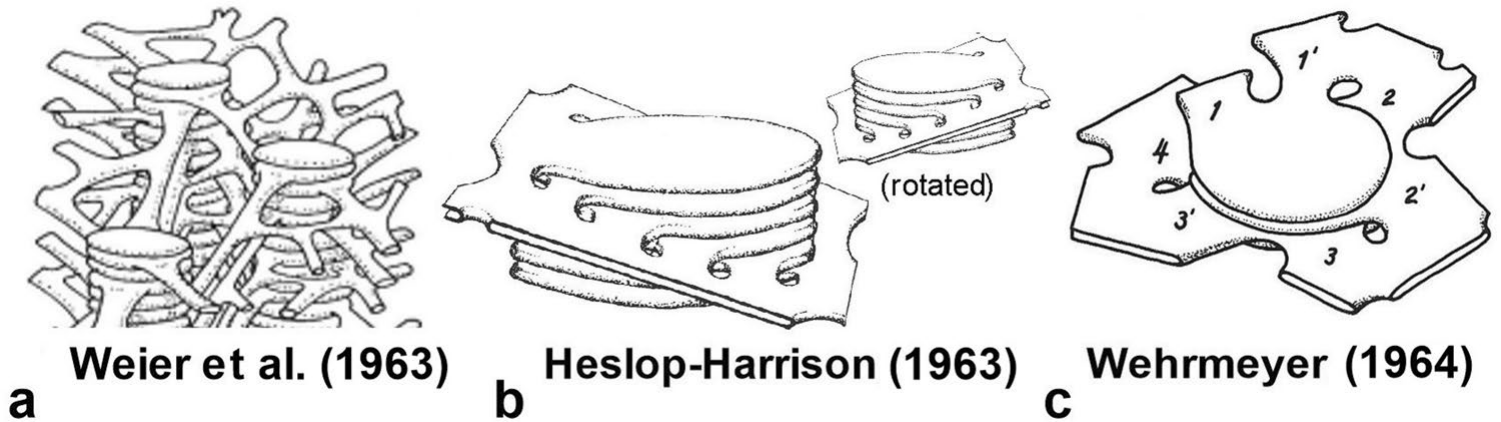
Three-dimensional chloroplasts models of the 1960s that replaced those proposed in the 1950s (Fig. 5). **a** The characteristic feature of the Weier et al. (1963) model is the much exaggerated tubularity of the stroma thylakoids. **b** The 1963 model shows a planar stroma thylakoid intercepting a cylindrical grana stack. The two images illustrate the front and back sides of the model and the series of connections that connect the stroma thylakoid with the grana thylakoids. In this model, the partial helices formed by the stroma thylakoid are of opposite handedness on the near vs. far sides of a granum. **c** In the Wehrmeyer (1964) model the stroma thylakoid is depicted as a right-handed helix that spirals around the granum, while also suggesting a mechanism for spiral growth

The next phase of studies devoted to the elucidation of the 3D architecture of thylakoids involved careful analyses of the geometry of the grana margins. Weier and Thomson (1962) described ten different types of configurations at the margins of grana as seen in cross-sectional views. In 1963, Weier et al. (1963) summarized their data in the 3D model presented in Fig. 6a. This model depicts the grana as being connected to each other by tubules that extend in different directions. Not included in the model was the observation of Weier and Thompson (1962) that stroma thylakoids were connected to grana thylakoids in a helical manner. The Weier et al. (1963) model was criticized for overemphasizing tubularity of the stroma thylakoids by Heslop-Harrison (1963) and by Wehmeyer (1964), because their observations indicated that stroma thylakoids are fenestrated (perforated) sheets as opposed to arrays of tubules.

After Falk and Sitte (1963) reported that potassium permanganate transformed lamellar thylakoid sheets into tubules and similar findings were observed by Wehrmeyer (1964), several laboratories embarked on systematic studies of the how different fixatives (osmium tetroxide, glutaraldehyde, potassium permanganate), different buffers, different dehydration protocols, and different resins and growth conditions affected the structure of chloroplasts. According to Paolillo et al. (1967), the most important parameter for obtaining reproducible results with different fixatives was the degree of the intactness of the chloroplasts. Damaged chloroplasts exhibited much greater structural variability. Differences in the amount of fenestration in different species were also noted, with tobacco, hemp and spinach thylakoids having more fenestrae than elodea, pea, bean, and corn.

The model developed by Heslop-Harrison (Fig. 6b; 1963) shows a planar stroma thylakoid intercepting a cylindrical granum at an angle with helical connections on both sides of the granum (see back and front views in Fig. 6b). However, with this model the two partial helices so created would have opposite tilts, if left-handed on the near side of the granum (as in the original figure) then right-handed on the far side. The first models showing helically arranged stroma thylakoids wrapping around and connected to the stacked grana thylakoids were published in 1964 (Fig. 6c).

**Fig. 7:**
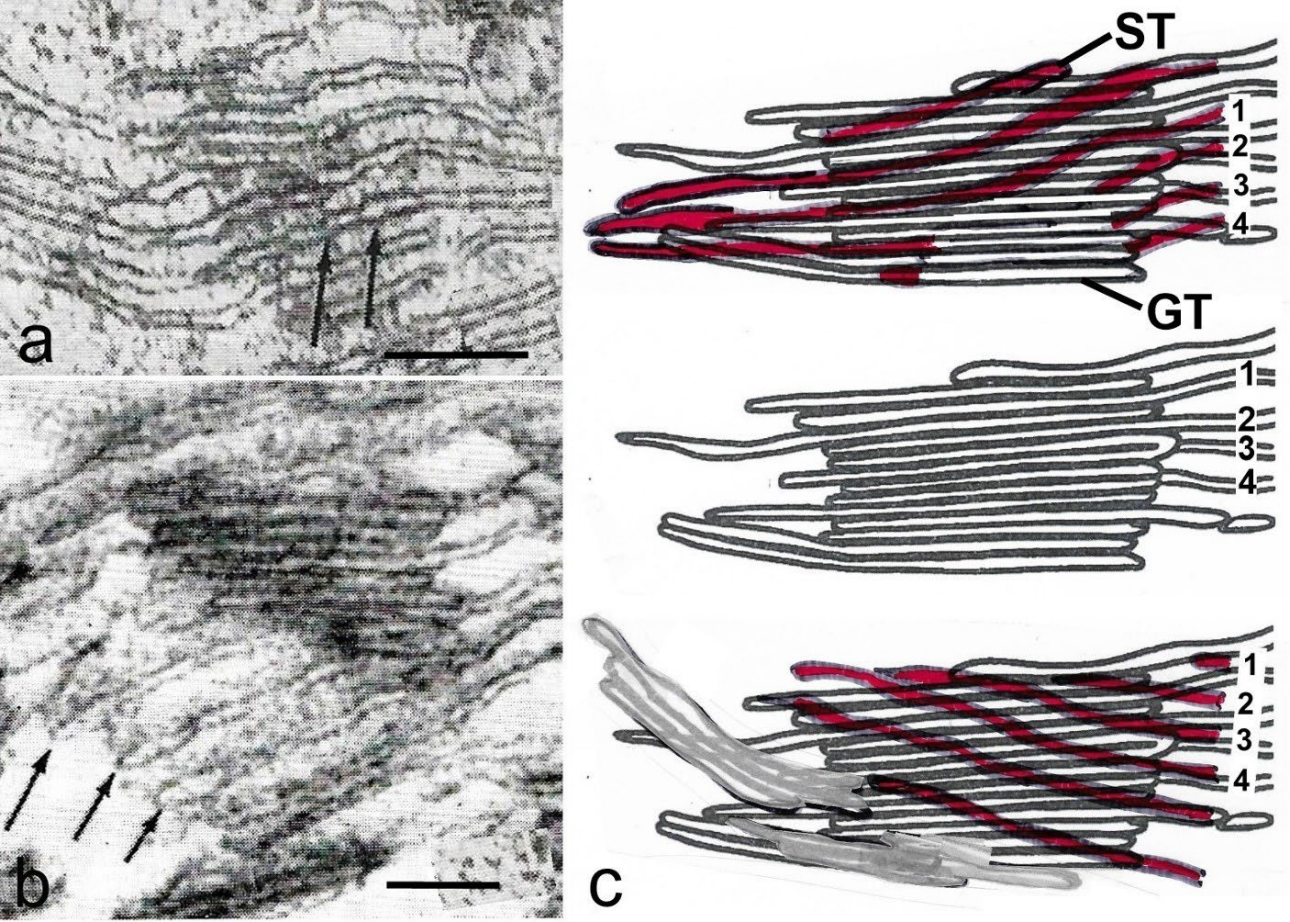
Tangential sections and tracings of serial sections through a grana stack. **a** The non-stacked stroma thylakoids are angled with respect to the stacked grana thylakoids and show evidence for a step-like ascent (arrows) corresponding to the frequency of the grana thylakoids. **b** Three evenly spaced stroma thylakoids marked by arrows appear angled to the plane of the stacked grana thylakoids in both the plane of the section and in the third dimension. **c** Tracings from three selected sections in a series of ten serial sections of a granum and associated stroma thylakoids from a bean chloroplast. The median section is reproduced in all three images, with the tangential sections from the near side (uppermost image) and far side (lowermost image) of the granum superimposed upon it. Stroma thylakoids in the tangential sections are shown in red. The helical structure of the stroma thylakoids can be inferred within a series of sections by comparing the tilt of the membranes as seen on the near versus the far tangents. Stroma thylakoids that could be followed completely around the grana stack are numbered (1–4). **a**, **b** From Paolillo and Falk (1966); **c** based on Figs. 1–10 in Paolillo and Reighard (1967); **a, b** Bars 0.1 mm

Wehrmeyer’s (1964) model arose from his investigations of how grana stacks were formed during chloroplast development. Working with immature chloroplasts he demonstrated the concept that a single granum thylakoid could generate an outgrowth that overtopped the first granum thylakoid to form a new grana stack. Repetition of the membrane outgrowth and expansion process leads to an increase in height of a granum stack and to spiral-cyclical growth around the granum (Fig. 6c). A more detailed analysis of the generation of these outgrowths was subsequently published by Brangeon and Mustardy (1979) who showed that the rims of the fenestrae in the expansive proplastid thylakoids were the sites where formation of the stacked membrane regions is initiated with the formation of a membrane tongue that overgrows the forming membrane and starts the spiral growth. For mature chloroplasts, Paolillo and Falk (1966) demonstrated that oblique stroma thylakoids wind around each granum as continuous helices, and they proposed that the observed spacing between the slanted stroma thylakoids in tangential views of the grana (Figs. 4b, 7a–c) is consistent with the grana being surrounded by multiple helices.

**Fig. 8:**
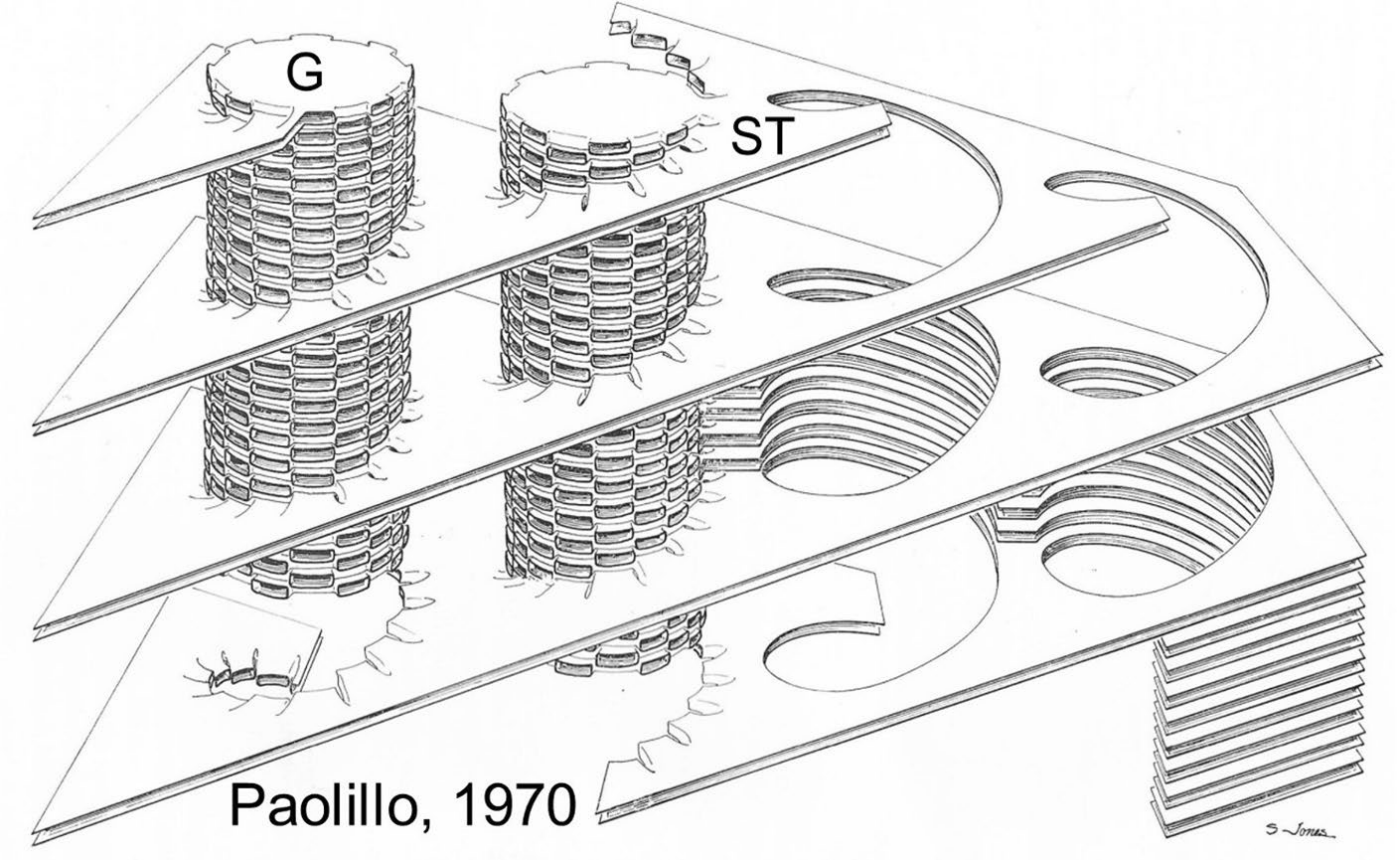
Three-dimensional model depicting how the grana thylakoids are connected to the stroma thylakoids. The stroma thylakoids (ST) are shown to be organized as parallel sheets that form right-handed helices around each granum (G). On the right side, two grana are omitted to illustrate eight thylakoids that spiral around the grana stacks. At the left, seven of the eight stroma thylakoids were omitted so that the numerous connections between grana and stroma lamellae could be drawn in. From Paolillo (1970)

The helical model (Fig. 8) was generalized by showing that the data from seven species of plants commonly used for electron microscopy of chloroplasts fit the model (Paolillo et al. 1969). However, unresolved by the model was the question whether all of the helices within a plastid are codirectional, and if so, whether they are left- or right-handed. This question was settled by direct reconstruction of numerous grana from serial sections, like those in Fig. 7c, but where the absolute orientation of the sections was also known because the blocks were trimmed asymmetrically (Paolillo 1970). The helices were found to be right-handed (ascending to the right at the near tangent of a grana stack). The handedness of the helices was confirmed for chloroplasts in five species of plants.

Brangeon and Mustardy (1979) constructed a refined helical model that filled in some of the details missing from the earlier Paolillo (1970) study. Most notably they provided information on the dimensions of the slits in the grana thylakoids that attach them to the stroma thylakoids, and the dimensions of the closed portions of the grana that are free of connections. Using scanning electron microscopy Mustardy and Janossy (1979) provided an image of an isolated granum encompassed by helical membranes. Other, findings of reconstructions of serial sections are reviewed in Mustardy (1996), Mustardy and Garab (2003), and Mustardy et al. (2008).

Wehrmeyer's (1964) concept of spiral-cyclical growth of grana stacks indicates that the helical structure is present at the periphery of a granum from its earliest stage of formation (see also Mustardy 1996; Liang et al. 2018; Kowalewska et al. 2019). During the later stages of chloroplast development, additional stroma thylakoid helices are added to the initial one, either by splitting of the initial helix, or by conjoining of a grana stack with a ramification of a nearby stroma thylakoid (Brangeon and Mustardy 1979; Mustardy 1996).

**Fig. 9:**
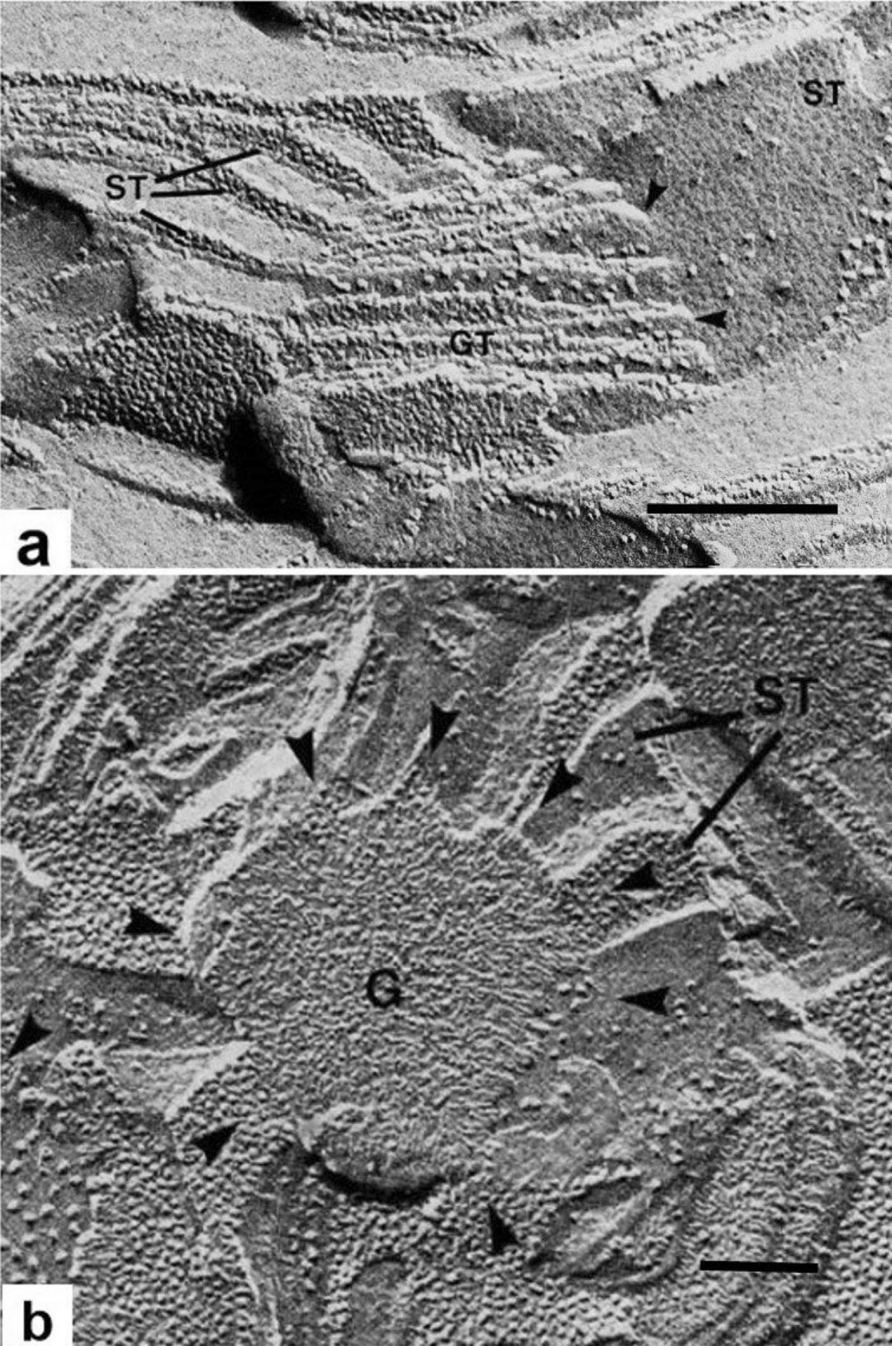
Freeze-fracture electron micrographs illustrating the 3D relationship between stroma thylakoids and their associated grana thylakoids. **a** The grana thylakoids (GT) in the center are fractured obliquely, while the stroma thylakoid (ST) on the right is seen in a face-on view. Two of the junctional connections between the grana and the stroma thylakoids are marked by arrowheads. On the left side, several parallel stroma thylakoids are cross-fractured. All of the structural features of the membranes seen in this micrograph are consistent with multiple helical stroma thylakoids wound around the granum as postulated by the helical model. **b** Face-on view of a grana (G) stack with angled stroma thylakoids (ST) connected to its margins (arrowheads) and arranged like the blades of a conventional windmill with the central granum corresponding to the hub. The image also supports the helical model. **a, b** From Staehelin and van der Staay (1996). **a, b** Bars 0.2 mm

As illustrated in Fig. 9, freeze-fracture EM (for technical details about this technique see next section) provided direct support for the helical model (Fig. 8) by visualizing grana and stroma thylakoids in both face-on and cross-sectional views. In Fig. 9a, the stroma membrane is seen in a face-on view and the grana thylakoids, to which it is connected through short, tubular junctions, are seen in oblique views. The helical nature of the stroma thylakoid is clearly seen. In contrast, Fig. 9b provides face-on views of a round grana thylakoid to which stroma membranes are attached at an angle in a windmill-type manner. Another feature of Fig. 9b is the difference in particle composition and distribution between the grana and stroma membrane fracture faces. As discussed in the next section in greater detail, the particles correspond to protein complexes of the photosynthetic electron transport chain, and their segregation patterns reflect functional differences between the photosystem II (PSII)-rich grana and the photosystem I (PSI)-rich stroma membranes (Staehelin 2003). In the words of Trissl and Wilhelm (1993), grana stacking is one of Nature's structural mechanisms for the separation of PSI and PSII, thereby preventing direct competition between the faster PSI and the slower PSII reaction center complexes.

Grana membrane stacking is a reversible process that can be controlled in in vitro experiments by changing the concentration of Mg or Na ions in the suspending medium (Izawa and Good 1966). This result was confirmed by Staehelin (1976), who further demonstrated that unstacking in a low salt buffer led to an intermixing of the PSII and PSI particles, while restacking and re-segregation occurred when Mg^2+^ or Na^+^ were added. Thus, it appears that the membranes of mature chloroplasts retain the capability to form and reform stacked grana regions. In leaves, unstacking and restacking of grana thylakoids occurs during State I and State II transitions, and is controlled by the phosphorylation of LHCII and PSII proteins (Kyle et al. 1983; Staehelin and Arntzen 1983; Kirchhoff et al. 2007; Anderson et al. 2012; Wood et al. 2018).

**Fig. 10:**
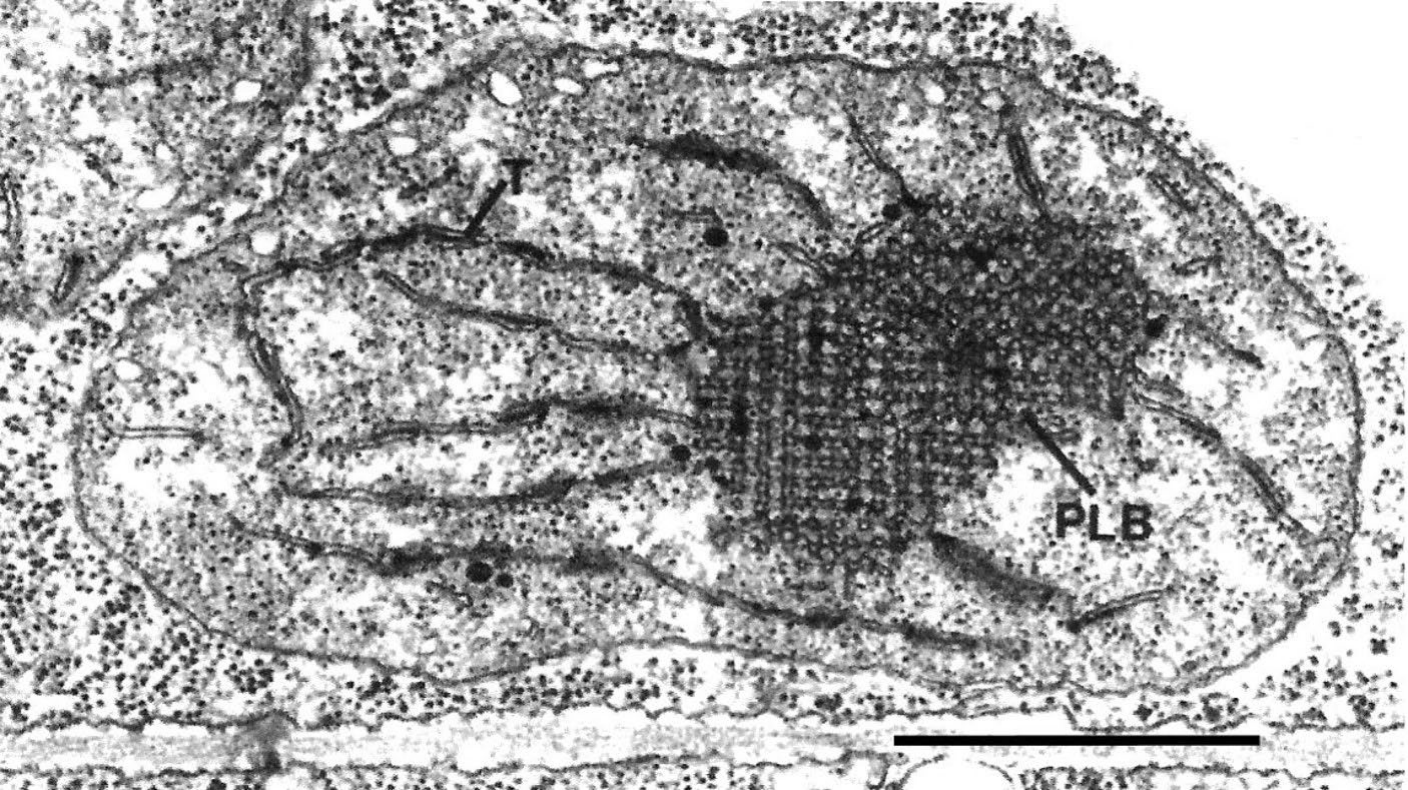
Etioplast of a chemically fixed pea cell with a large, well-organized prolamellar body (PLB), typical of seedlings kept for prolonged periods of time in darkness prior to exposure to light. Thylakoids (T) are attached to the margins of the PLB. From Staehelin (1986). Bar 0.3 mm

The structure of thylakoid membranes is dependent on the light regime in which the plant grows. Etiolated (light deprived) plants have been used extensively to investigate structural, biochemical and biophysical aspects of chloroplast development (Adam et al 2011). Etiolated plants can be created experimentally by growing them in the dark or can arise naturally when they grow underground after germination. The plastids of etiolated plants are known as etioplasts. They produce characteristic, 3D paracrystalline tubular networks called prolamellar bodies (PLBs) composed primarily of chloroplast lipids, protochlorophyllide, and the light-dependent complex protochlorophyllide oxidorreductase (Fig. 10; Gunning 1965; Weier and Brown 1970; Weier et al. 1970; Solymosi and Schoefs 2010; Kowalewska et al. 2016). In the dark only pro-thylakoid stubs are seen as being continuous with marginal PLB tubules. Upon exposure to light and the conversion of protochlorophyllide to chlorophyllide the pro-thylakoid membranes expand at the expense of the PLBs while being converted into photosynthetically active thylakoids. Because many environmental parameters can be controlled in this system, it has been widely used for studies of thylakoid assembly. Plastoglobuli (Figs. 3, 4b) that accumulate in etiolated plastids also become diminished when exposed to light, suggesting that, like the prolamellar bodies, they contribute to the formation of the developing chloroplast membranes (Lichtenthaler 2013). Chloroplasts that mature after etiolation are structurally normal (Rudowska et al. 2012).

## Atomic force microscopy, freeze-fracture/etch electron microscopy, and electron tomography have produced macromolecular information about thylakoid membranes

This section focuses on how new techniques introduced since 2000 to photosynthesis research have led to important novel insights into the structure of thylakoids, and on how some of those discoveries relate to previously published freeze-fracture/etch findings. The new techniques include atomic force microscopy to investigate the macromolecular organization of thylakoids in their hydrated state, and electron tomography (ET) to analyze the structure of cryofixed chloroplasts and to create both high-resolution 3D models of thylakoid architecture and information about the macromolecular organization of thylakoid membranes.

The main advantages of electron tomography and atomic force microscopy over conventional transmission electron microscopy is their higher *x*-, *y*- and *z*-axis resolution. Conventional electron microscopy using 60–80 nm thick plastic sections has an *x*/*y*-axis resolution of ~ 5 nm and a *z*-axis resolution of 12–16 nm. In contrast, atomic force microscopy and electron tomography have a resolution that is over 10 times better. This allows for the reconstruction of much higher resolution 3D models, and for more detailed analyses on the organization of specific complexes in the plane of the membranes.

### Dual-axis electron tomography (ET)

The greatly improved 3D resolution of biological structures imaged by dual-axis ET is primarily due to its improved *z*-axis resolution (McIntosh et al. 2005; Donohoe et al. 2006). This is done by combining multiple 2D images of objects as they are systematically tilted from + 60° to − 60° along two orthogonal axes. The selected 3D structure is computed by back-projecting each 2D image with appropriate weighting (Koster et al. 1997). The resulting 3D block of data points are called voxels (volume elements), each of which is assigned a mass density. The voxel information can be displayed as 2D images of selected *z*-axis levels that resemble thin sections, except that they are only 2 nm thick, versus 60–80 nm for thin sections. This results in a substantially improved resolution of 3D reconstructions of cellular structures.

### Cryofixation methods

To take full advantage of the high resolution of ET, it is imperative that the samples are processed for viewing as close as possible to a life-like state. This can only be achieved by means of cryofixation methods with a freezing rate of at least − 1000 °C/ms (Gilkey and Staehelin, 1986). Under these conditions, the samples become vitrified, with all types of cellular molecules and structures being immobilized simultaneously. The two cryofixation methods used by electron microscopists today are plunge freezing (Dubochet et al. 1988) and high pressure freezing (Kang 2010). Chemical fixatives, on the other hand, are limited in their ability to preserve cells in their natural state, because each fixative acts by crosslinking a subset of molecules (Gilkey and Staehelin 1986), e.g., glutaraldehyde only crosslinks proteins but not lipids, and these reactions take minutes to fully immobilize cells (Buckley 1965). For this reason, chemical fixation yields less reliable images of cellular structures than cryofixation, which, in turn, reduces the resolution of the electron tomogram images.

### Electron tomography studies of cryofixed and plastic-embedded samples

The first ET study of cryofixed and plastic-embedded thylakoids led to the formulation of the flawed “bifurcation” model (Shimoni et al. 2005). This model was flawed due to its inability to explain all of the data that support the helical model (see previous section). This problem was caused by the fact that the study only involved the analysis of single thin sections that lacked *z*-axis depth to discern the helical membranes in 3D reconstructions.

**Fig. 11:**
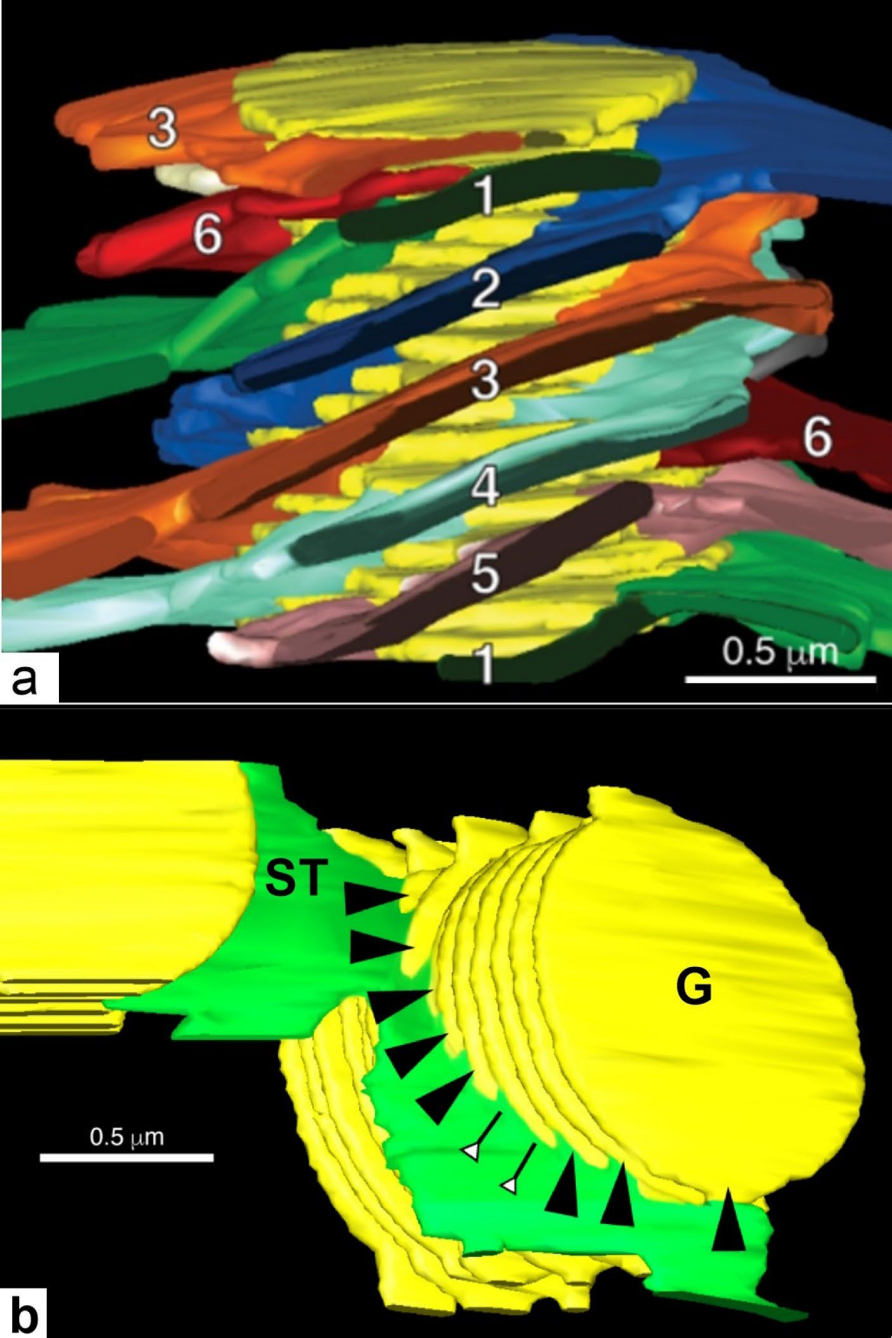
Electron tomography-based model of a grana stack and associated stroma thylakoids (ST) of an Arabidopsis chloroplast. **a** The grana thylakoids are colored yellow, and the stroma thylakoids are colored differently and numbered to allow for tracking their position in different views of the model (not shown). **b** Electron tomography model of two grana stacks (G, yellow) and a green colored stroma thylakoid that forms both a link between the two stacks and illustrates how stroma thylakoids spiral around and interact with grana stacks. The black arrowheads point to the typical, smaller size grana-stroma junctions that the helical stroma thylakoid forms with each grana thylakoid. The white arrowheads mark a stroma-grana membrane junction with double the width of the regular ones. Such large junctional slits arise when two junctions are formed adjacent to each other on a single grana thylakoid. **a**, **b** From Austin and Staehelin (2011) Copyright American Society of Plant Biologists

In contrast, the ET analysis of entire grana stacks and connecting stroma thylakoids by Austin and Staehelin (2011) yielded reconstructions that fully support the defining features of the helical model. Their 3D reconstructions demonstrated that the stroma thylakoids were wound around the grana stacks in parallel, right-handed helices, with the number of helical stroma membranes being variable (Fig. 11a). The angle was 20° to 25°, the same as reported in several previous studies (Paolillo 1970; Mustardy et al. 2008), but higher than the 15.9°–18.4° measured by others (Bussi et al. 2019). The different angles most likely reflect the variability of chloroplasts in plants grown under different light conditions.

The most important difference with the Paolillo (1970) helical model was the greater variability of the width of the junctional connections between the grana and stroma thylakoids, which was 30 nm to 400 nm versus ~ 70 nm as determined by Mustardy et al. (2008) and 60 to 100 nm by Bussi et al. (2019). In the Austin and Staehelin (2011) study the extra wide junctions were seen where a given stroma thylakoid had formed two adjacent but merged junctional connections with the same grana thylakoid instead of with adjacent thylakoids of the stack (Fig. 11b). The variability of the size of the junctions suggests that regulation of their size may serve as a mechanism for regulating thylakoid activities. Considering the high rates of lateral diffusion of thylakoid proteins and lipids (Kirchhoff et al. 2008a; Kirchhoff 2014), the size of the junctions should not impede the rapid exchange of the protein complexes between grana and stroma membrane regions during state 1–state 2 transitions.

**Fig. 12:**
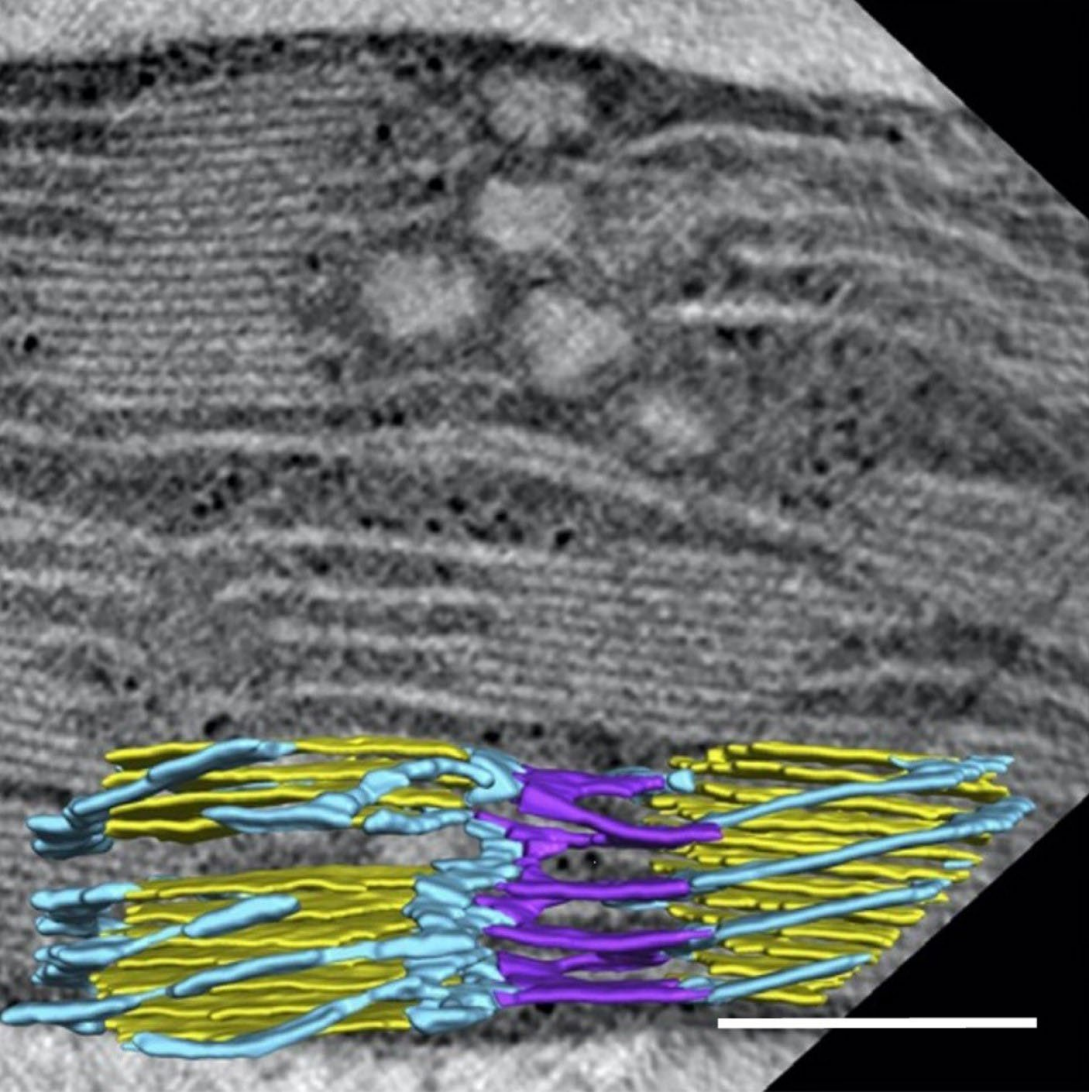
Electron tomography slice image of a lettuce chloroplast illustrating stacked grana thylakoids and non-stacked stroma thylakoids. Superimposed in the lower part of the micrograph is a 3D tomographic model that illustrates how two sub-groups of stroma thylakoids (blue, purple) bridge the space between two grana stacks (yellow). Thus, while the stroma thylakoids that form connections to the grana thylakoids are organized as right-handed helices (blue), those that connect the two sets of stroma thylakoids around the grana stacks are linked to each other through left-handed helices (purple). From Bussi et al. (2019). Bar 0.2 mm

A detailed analysis of the 3D architecture of the grana and stroma thylakoids in entire, cryofixed chloroplasts by means of several different ET and specimen preparation techniques has provided important information on how the stroma thylakoids are organized between grana stacks (Bussi et al. 2019). The novel information to come from this investigation was that the stroma thylakoids are arranged in both right- and left-handed helices, with membrane bifurcations mediating the transitions between the two helical types (Fig. 12). This balance of right- and left-handed helices minimizes the surface and bending energies of the membranes.

**Fig. 13:**
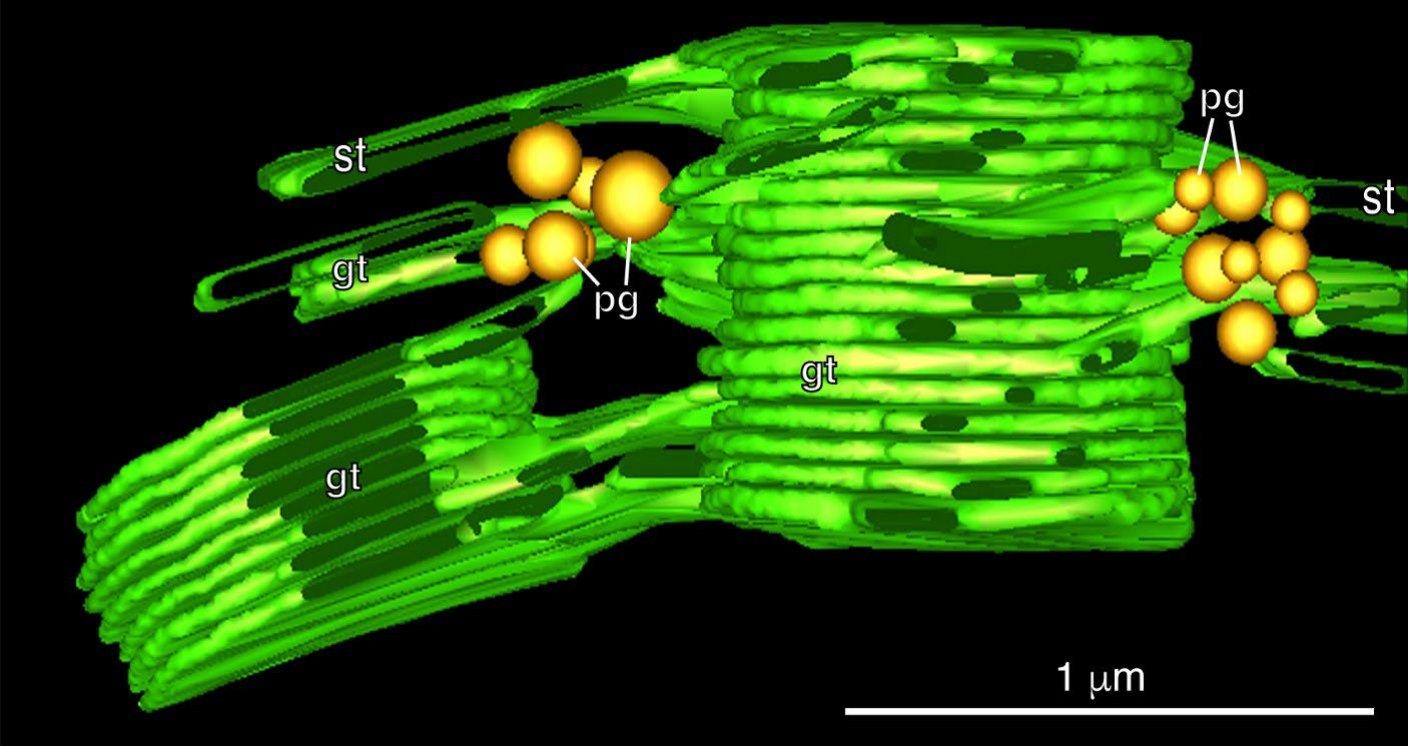
Tomographic model depicting two clusters of plastoglobules (pg) that are closely associated with thylakoid membranes in an Arabidopsis chloroplast. How the plastoglobules are connected to thylakoid membranes and to each other is depicted in greater detail in the diagram Fig. 14. Grana thylakoids (gt) and stroma thylakoids (st). From Austin et al. (2006). Copyright American Society of Plant Biologists

**Fig. 14:**
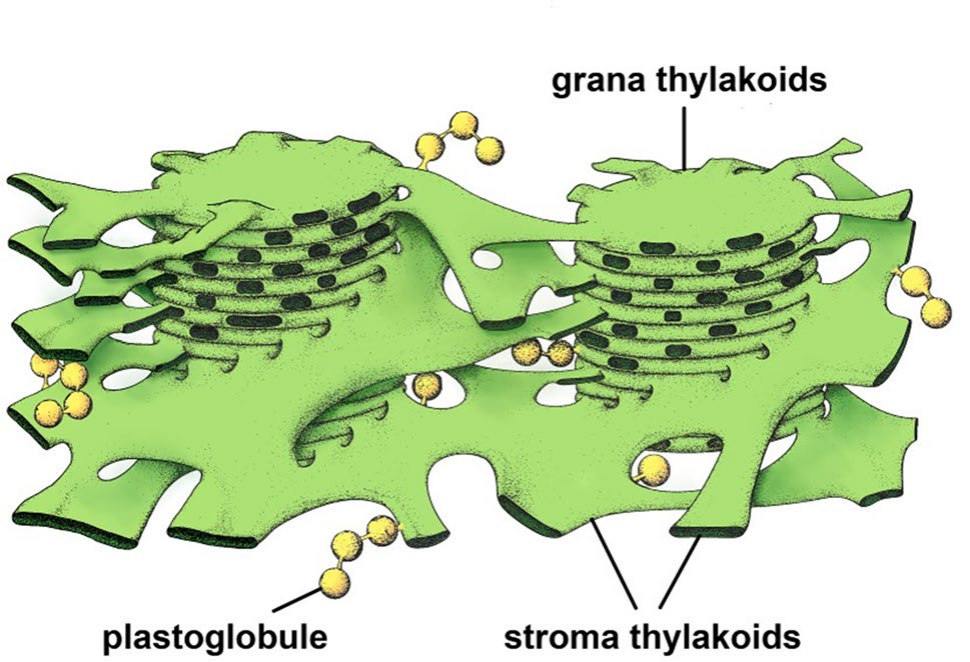
3D model of thylakoid membranes with bound plastoglobules based on electron tomography reconstructions of cryofixed and plastic-embedded chloroplasts. The stroma thylakoids that spiral around the grana stacks form right-handed helices as postulated by the helical model. While some of the grana-connecting stroma thylakoids form right-handed helices, some fork to create left-handed helices. The plastoglobules are surrounded by and linked to each other and to the thylakoid membranes via a lipid monolayer that is continuous with the stroma leaflet of the thylakoid membrane bilayer

Plastoglobules (Figs. 3, 4) have been known since the onset of electron microscope studies of chloroplasts. They were typically viewed as stroma-localized globules that sometimes appeared to be associated with thylakoid membranes (Lichtenthaler 2013). This view changed with the analysis of plastoglobules of cryofixed chloroplasts embedded in plastic (Fig. 13; Austin et al. 2006). Their analysis demonstrated that plastoglobules are permanently attached to thylakoids through a half-lipid bilayer that surrounds the globule contents and is continuous with the stroma leaflet of the thylakoid membrane. The half-lipid bilayer encompassing the neutral lipidic contents (carotenoid esters, tocopherols, plastoquinone and phylloquinone) contains ~ 30 core structural and enzymatic proteins, with the latter involved in lipid metabolism (Van Wijk and Kessler 2017). A diagram illustrating the 3D architecture of higher plant thylakoids with attached plastoglobules is presented in Fig. 14.

Novel insights into how the intricate 3D thylakoid networks are assembled during *Arabidopsis* seedling development have been gained by combining ET, gene expression and mutant analysis of developing chloroplasts (Liang et al. 2018). By germinating the seedlings under constant illumination, the authors were able to create an experimental system where the development occurred in a sequential manner with the photosynthetic membrane complexes appearing sequentially over a five-day period. In turn, this experimental system has provided a means for correlating the incorporation of specific proteins into the developing thylakoids with specific structural membrane changes. Twenty-four hours after incubation (HAI) the proplastids contain tubulovesicular membranes that form contacts along their margins with the inner envelope membrane. Such contact sites are seen throughout development as well as in mature chloroplasts. After 36 h, flattened pre-grana thylakoids are formed by the binding of polysomes that appear to insert core proteins of PSII complexes. At 60 HAI pre-grana stacks begin to be formed and grow into larger separate stacks at the sites of polysome binding. Simultaneously LHCII, cytb6f and ATP synthase polypeptides can be detected in the membranes. The grana-stroma thylakoid network begins to be formed at 84 HAI as the separate grana stacks are joined laterally together by FZL membrane fusion proteins, and as PSI polypeptides and curvature forming CURT1 proteins are inserted into the membranes. Mature grana-stroma thylakoid networks are seen 120 HAI.

### Freeze-fracture and freeze-etch techniques

**Fig. 15:**
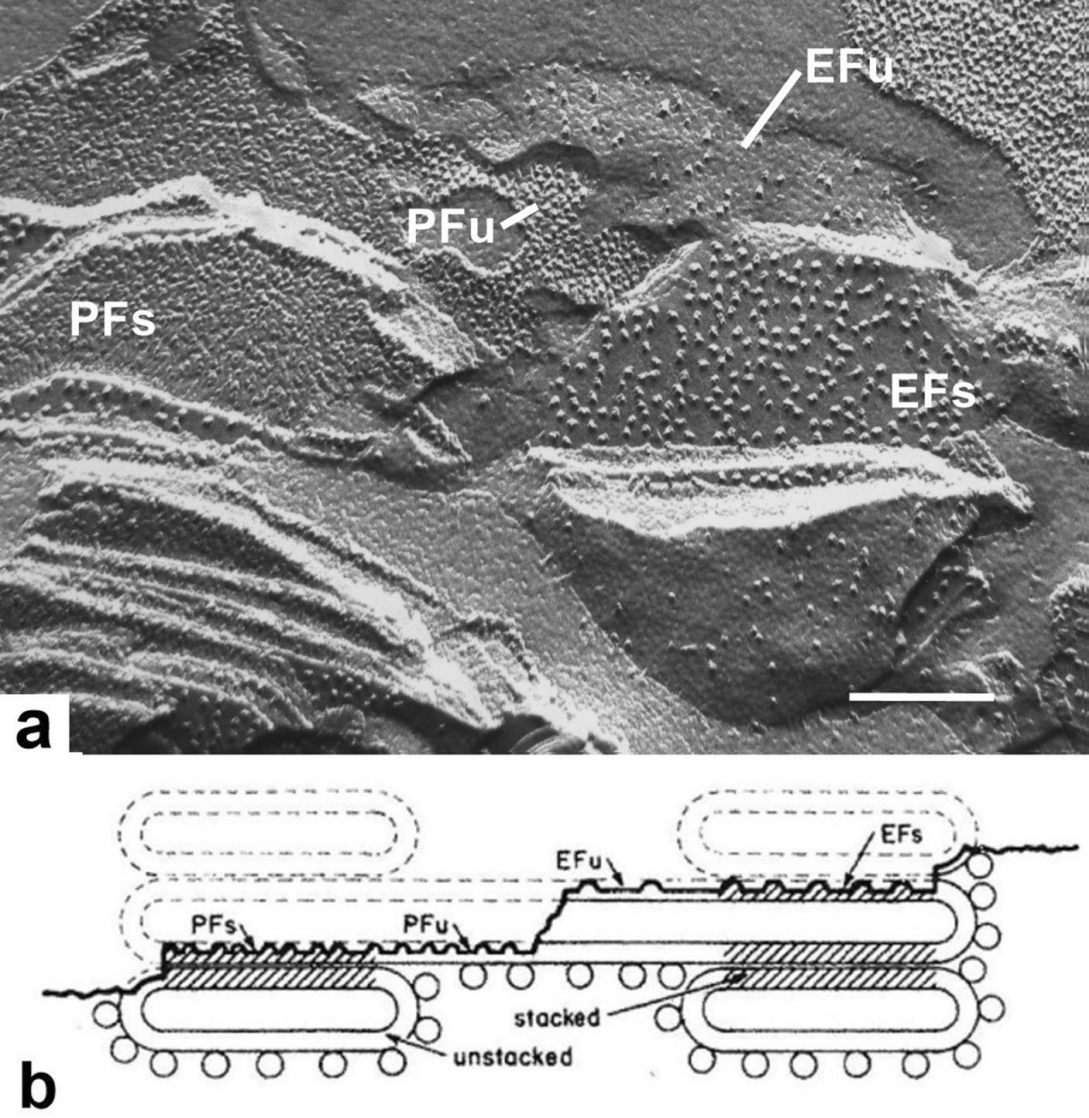
Freeze-fracture electron micrograph of spinach thylakoids. **a** The semi-circular structures on the left and the right correspond to fracture face views (labeled EFs and PFs) of grana thylakoids generated by splitting of the membrane bilayers as explained in (**b**). The fracture faces labeled EFu and Pfu belong to unstacked stroma thylakoids that connect the two adjacent grana stacks. The large EFs particles correspond to PSII-LHCII complexes, and the smaller EFu particles to incomplete PSII complexes. The PFs particles correspond to free LHCII and cytb_6_f complexes, whereas the PFu particles represent PSI, cytb_6_f, free LHCII and CF_0_ complexes. **b** Schematic diagram illustrating the nomenclature used to label freeze-fracture and freeze-etch images of thylakoid membranes (see **a** and Fig. 16a). From Staehelin and Arntzen (1983). **a** Bar 0.2 mm

The process of freeze-fracturing of frozen samples is carried out at − 100 °C in a high vacuum. After fracturing, high-resolution PT/C replicas are produced of the exposed fracture faces and, after cleaning, the replicas are viewed in a transmission EM. At − 100 °C, bilayer membranes are split during the fracturing of the samples along their central hydrophobic plane, while the integral protein complexes deflect the fracture plane, yielding particles of different size that protrude from the smooth surfaces of the split bilayers (Fig. 15a). The membrane splitting process produces two complementary fracture faces, known as the EF and PF (endoplasmic and protoplasmic) faces (Fig. 15b). Because each protein complex partitions to the side of the bilayer leaflet with the larger exposed hydrophilic domains, the EF and PF faces exhibit different sets of particles, with the PSII particles seen on EF faces and PSI, cytb6f, free LHCII and the CF0 domain of the ATP synthase on PF faces.

Correlation of the freeze-fracture particles with specific functional complexes was achieved through a combination of quantitative structural and biochemical studies of plants grown under different illumination conditions, the analysis of chloroplast mutants, and characterization of the particles produced by purified protein complexes reconstituted into lipid vesicles (reviewed in Staehelin and van der Staay 1996). Based on these criteria, the PSII complexes give rise to large EFs particles that are concentrated in the stacked grana membrane regions (Fig. 15a). Quantitative analysis of the distribution of these particles shows that ~ 85% are localized to the stacked membrane domains. Furthermore, the particles possess stepwise differences in size due to the number of attached LHCII complexes (Armond et al. 1977). Reconstitution of isolated cytb_6_f, CF_0_ and trimeric LHCII complexes into liposomes produced particle size information that helped identify such complexes in intact membranes (McDonnel and Staehelin 1980; Mörschel and Staehelin 1983). These studies also demonstrated that reconstituted LHCII complexes can mediate membrane adhesion in the presence of Na and Mg ions, and that cytb_6_f is a dimeric complex.

**Fig. 16:**
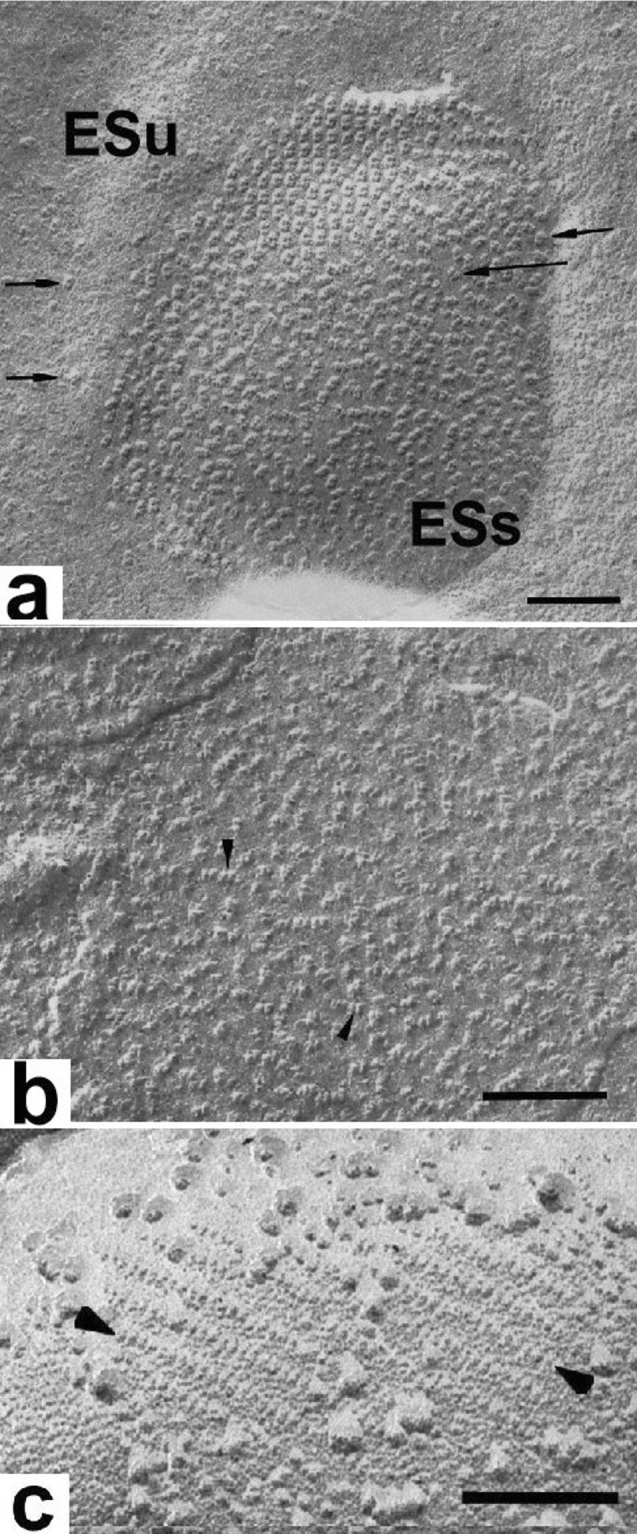
**a** Luminal surface view of a spinach thylakoid exposed by freeze-etching. A central, dimeric particle-rich grana domain (ESs) is surrounded by a stroma thylakoid domain (ESu) with few dimeric particles (arrows). The dimeric particles represent the protruding parts of the oxygen-evolving complexes of the dimeric PSII/LHCII complexes. Some of the dimeric particles form a small lattice. **b** The dimeric particles (arrows) seen on the luminal surface of a grana membrane after removal of the oxygen-evolving proteins correspond to the core PSII/LHCII complexes, but are smaller than those seen in (**a**). **c** Stroma surface of an experimentally unstacked grana thylakoid with arrayed PSII particles (arrows). Together **b** and **c** demonstrate that the PSII complexes are integral membrane complexes that extend across the bilayer and protrude from both sides of the bilayer membranes. **a** From Staehelin (1976), **b** from Seibert et al. (1987), **c** from Miller (1976). **a** Bars 0.1 mm, **b** 0.1 mm, **c** 0.2 mm

Freeze-etch electron microscope studies are used to analyze the organization of peripheral proteins associated with the integral proteins as well as the surface domains of the integral protein complexes (Staehelin 2003). Figure 16a illustrates the organization of the PSII water-splitting enzymes on the luminal surface (ESs surface) of the grana thylakoids. While most of the complexes show a random organization, a small group is organized in a lattice (such lattices are atypical but seen more often in spinach samples purchased in winter months). The water-splitting enzymes exhibit a dimeric structure, which reflects the dimeric nature of the underlying PSII core complexes. The luminal surface of the latter complexes can be visualized after removal of the water-splitting enzymes (Fig. 16b; Seibert et al. 1987). Imaging of the minimally protruding stromal surface domains of the PSII complexes requires experimental unstacking of the grana membranes before etching and replication (Fig. 16c; Miller 1976). In other reconstitution experiments, the surface of the LHCII complexes was shown to barely extend beyond the bilayer surface (McDonnel and Staehelin 1980). The main limitation of the freeze-fracture/etch techniques is the resolution of the replicas when compared to the images obtained by atomic force microscopy and cryo-electron tomography (discussed below), and the fact that crystal structures of the protein complexes cannot be fitted into the replica images to identify the complexes. However, the thylakoid membrane areas that can be examined in freeze-fracture/etch micrographs are much larger than those visualized by the latter techniques.

### Atomic force microscopy (AFM)

The number of studies of the macromolecular organization of thylakoids using AFM is still rather limited in part because it has, in many cases, been superseded by cryo-EM studies (see below). Nevertheless, AFM is unique, because it is capable of displaying the surface topology of protein complexes of hydrated membranes (Kirchhoff et al. 2008b; Johnson et al. 2011; Wood et al. 2018). Its principal limitation is that it is only capable of producing high-resolution images of membrane samples that are firmly mounted on a mica surface, which requires pre-treatment of isolated thylakoids with digitonin. In the concentrations used, digitonin is a relatively gentle detergent that disrupts stroma thylakoids but leaves the grana membranes with small stroma membrane fragments largely intact (Dunahay et al. 1984). Digitonin-treated grana membrane fractions have been used in all AFM thylakoid studies published to date.

**Fig. 17:**
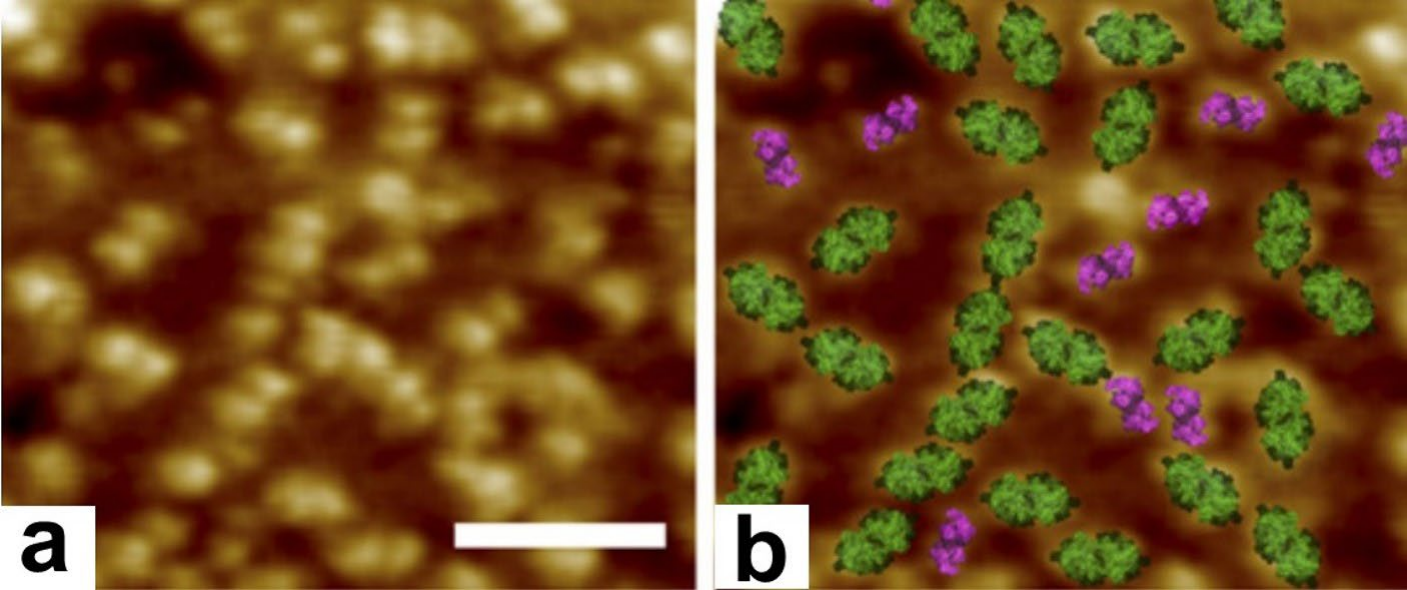
Identification of PSII and cytb_6_f complexes on the luminal surface of grana thylakoids in an AFM micrograph. **a** The AFM topographic image displays the membrane extrinsic parts of PSII and cytb_6_f complexes on the luminal surface of a grana thylakoid. **b** Identification of the complexes seen in (**a**) based on affinity mapping of cytb_6_f (purple) and crystal structure fitting of PSII (green) and cytb_6_f. From Johnson et al. (2014), Copyright American Society of Plant Biologists. Bar 50 nm

The report of Kirchhoff et al. (2008b) demonstrated convincingly that AFM could not only confirm the data produced by freeze-etch EM but also provide more high-resolution information on the molecular organization of thylakoid membranes. To identify the protein complexes of thylakoid membranes seen in AFM images, Johnson et al. (2011) employed two approaches, (1) they attached “bait” molecules to the AFM probe and then measured the strength of its binding to different membrane proteins, and (2) they fitted atomic structures of the membrane complexes into topological features they resolved in their micrographs. Together, these methods provided novel insights into the spatial relationship of the dimeric PSII and the cytb_6_f complexes in isolated and digitonin-treated grana membranes. Figure 17a shows an example of a high-resolution micrograph of the protruding luminal surface structures of a grana membrane, and Fig. 17b the experimentally identified dimer (green) and the cytb_6_f (purple) complexes.

By combining AFM, conventional EM and diverse biochemical and spectroscopic methods, Wood et al. (2018) have determined how the architecture and macromolecular organization of thylakoids are altered when spinach plants are transferred from dark to light. In conventional EM micrographs the transfer is seen to decrease the diameter of the grana while increasing grana numbers, two changes that increase the lateral contact areas between grana and stroma thylakoids. The complementing AFM data revealed that light causes the PSII complexes to become more widely spaced in the grana, while also increasing the spacing between the PSI complexes in the stroma thylakoids.

### Cryo-electron tomography

Cryo-ET can also be used to map the different kinds of protein complexes in ice-embedded membranes with great precision. The potential of cryo-ET for thylakoid membrane studies was first explored by Daum et al (2010) in a study focused mostly on PSII and ATP synthase complexes. Two experimental approaches were used for preparing the samples for cryo-ET analysis, thin sections of vitrified intact spinach and pea chloroplasts, and plunge frozen samples of isolated thylakoids. Due to the many technical problems associated with sectioning of the frozen chloroplasts, most of the study was directed towards the analysis of the isolated thylakoids. The latter experiments confirmed the dimeric nature of the PSII complexes in the grana thylakoids as well as the lollipop shape of the CF_1_ domain of the stroma membrane localized ATP synthases.

**Fig. 18:**
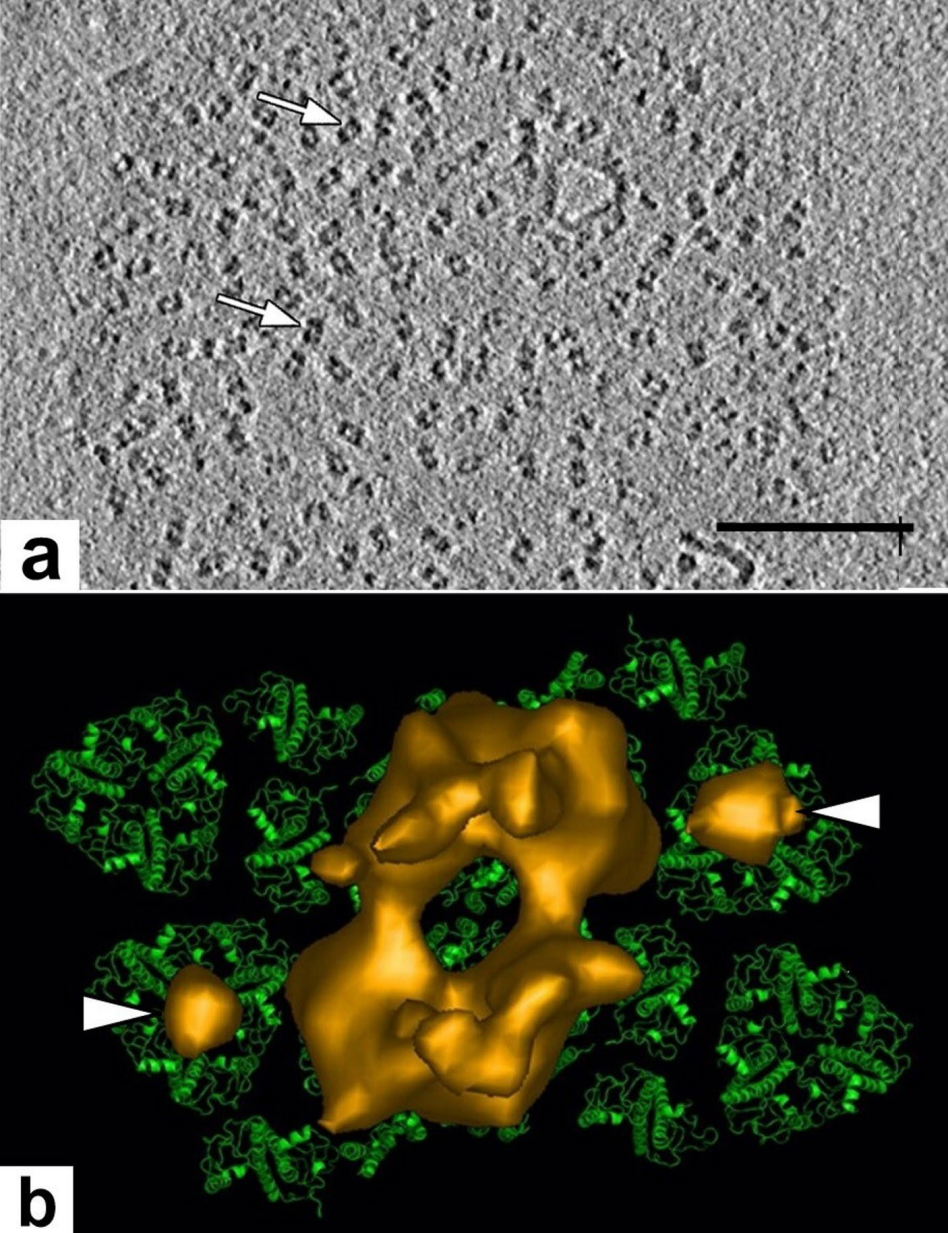
Cryo-ET slice image of a grana membrane and an isosurface model of a dimeric PSII complex of spinach. **a** The dimeric particles (arrows) correspond to protruding parts of PSII/LHCII complexes. **b** Isosurface model (brown-gold) that shows the extrinsic subunits of the oxygen-evolving complexes of a dimeric PSII complex on the luminal membrane surface superimposed on a pseudo-atomic model of a PSII-LHCII super-complex. The position of the two additional spherical densities (white arrowheads) coincides with the position of the S-type LHCII trimers and probably respresents violaxanthin. From Kouril et al. (2011). **a** Bar 0.1 mm

Kouril et al (2011) were able to refine these observations by examining grana of digitonin-treated isolated thylakoids. Their micrographs of the luminal membrane surface of grana membranes (Fig. 18a) provided clear images of the dimeric PSII core complexes together with their water-splitting proteins. By comparing the isosurface models with a pseudo-atomic model of the complete PSII super-complex C_2_S_2_M_2_ of PSII core complexes, the researchers were able to visualize not previously seen small particles, most likely violaxanthin deepoxidase proteins (Fig. 18b). According to their analysis, the position of these proteins coincides with the central coordinates of the PSII-bound LHCII complexes.

**Fig. 19:**
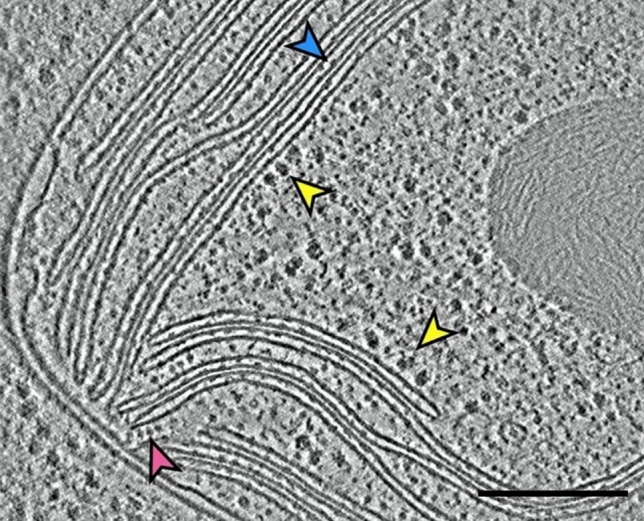
In situ cryo-ET slice through a chloroplast of an intact Chlamydomonas cell. The micrograph illustrates the native molecular architecture of both grana and stroma thylakoid and chloroplast envelope membranes. The arrowheads point to ATP synthases (red), the luminal domain of a PSII complex (blue), and membrane-bound ribosomes (yellow). The margins of several converging thylakoids appear to be attached to a translucent layer associated with the stroma surface of the inner envelope membrane. From Wietrzynski et al. (2020). Bar 0.2 mm

**Fig. 20:**
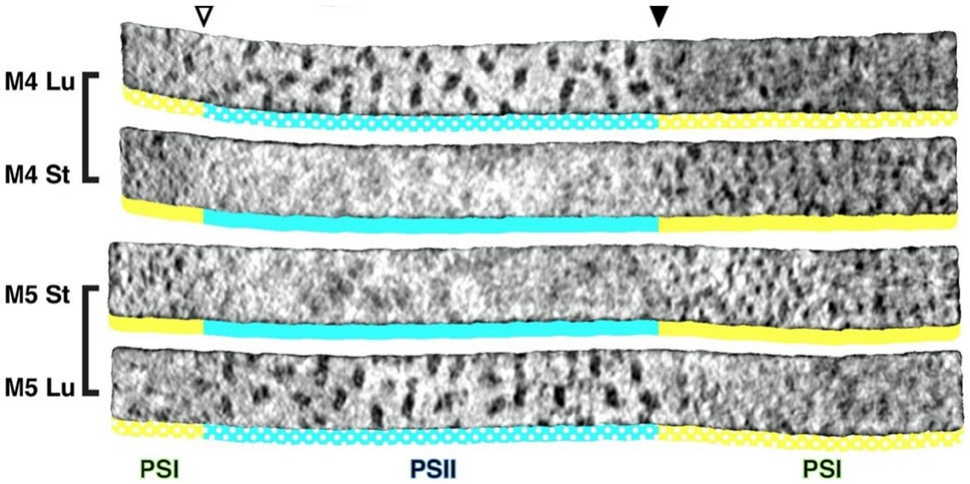
Membranograms reconstructed from cryo-ET images of PSII-rich stacked grana (blue) and PSI-rich non-stacked stroma thylakoids (yellow) of Chlamydomonas. All membranograms illustrate densities located 2 nm above the membrane surface. The stroma-side membrane surfaces are labeled St, and the luminal-side surfaces are labeled Lu. A sharp grana-to-stroma membrane transition (arrowhead) is evident in all membranograms. The large, dimeric PSII complexes are limited to the grana membrane domains, whereas the smaller PSI particles are limited to stroma membranes. From Wietrzynski et al. (2020)

The most recent advance of cryo-ET research comes from the combination of ion-milling, cryo-ET and improved image analysis methods (Engel et al. 2015; Wietrzynski et al. 2020). These studies have, for the first time, provided a means for obtaining in situ cryo-ET images of thylakoid membranes in vitrified *Chlamydomonas* cells (Fig. 19). The reason for including an ion-milling processing step after cryofixation was to produce, without damaging or distorting the cells, very thin frozen samples suitable for ET imaging in a cryo-EM. The images of the cross-sectioned thylakoid and envelope membranes are of exceptional clarity (Fig. 19). They also reveal distinct sites where the margins of the thylakoids are attached to the envelope membranes (also seen in higher plant chloroplasts as shown in Fig. 3), as well as clear images of the stroma-localized ribosomes. The same tomograms can also be used to characterize differences in distribution of protein complexes in the plane of the grana and stroma thylakoids (Fig. 20). The resolution of such micrographs is sufficient to fit crystal structure data to identify and characterize the individual complexes in the membranes. However, because the LHCII complexes do not protrude significantly above the surface of the membranes, their distribution can only be determined by indirect means. Nevertheless, the combination of techniques described in the Wietrzynski et al. (2020) paper has ushered in an exciting *new era* in thylakoid membrane research.
